# The *Edwardsiella piscicida* thioredoxin-like protein inhibits ASK1-MAPKs signaling cascades to promote pathogenesis during infection

**DOI:** 10.1371/journal.ppat.1007917

**Published:** 2019-07-17

**Authors:** Dahai Yang, Xiaohong Liu, Wenting Xu, Zhaoyan Gu, Cuiting Yang, Lingzhi Zhang, Jinchao Tan, Xin Zheng, Zhuang Wang, Shu Quan, Yuanxing Zhang, Qin Liu

**Affiliations:** 1 State Key Laboratory of Bioreactor Engineering, East China University of Science and Technology, Shanghai, China; 2 Shanghai Engineering Research Center of Maricultured Animal Vaccines, Shanghai, China; 3 Laboratory for Marine Biology and Biotechnology, Qingdao National Laboratory for Marine Science and Technology, Qingdao, China; Purdue University, UNITED STATES

## Abstract

It is important that bacterium can coordinately deliver several effectors into host cells to disturb the cellular progress during infection, however, the precise role of effectors in host cell cytosol remains to be resolved. In this study, we identified a new bacterial virulence effector from pathogenic *Edwardsiella piscicida*, which presents conserved crystal structure to thioredoxin family members and is defined as a thioredoxin-like protein (Trxlp). Unlike the classical bacterial thioredoxins, Trxlp can be translocated into host cells, mimicking endogenous thioredoxin to abrogate ASK1 homophilic interaction and phosphorylation, then suppressing the phosphorylation of downstream Erk1/2- and p38-MAPK signaling cascades. Moreover, Trxlp-mediated inhibition of ASK1-Erk/p38-MAPK axis promotes the pathogenesis of *E*. *piscicida* in zebrafish larvae infection model. Taken together, these data provide insights into the mechanism underlying the bacterial thioredoxin as a virulence effector in downmodulating the innate immune responses during *E*. *piscicida* infection.

## Author summary

Thioredoxin (Trx) is universally conserved thiol-oxidoreductase that regulates numerous cellular pathways under thiol-based redox control in both prokaryotic and eukaryotic organisms. Despite its central importance, the mechanism of bacterial Trx recognizes its target proteins in host cellular signaling remains unknown. Here, we uncover a bacterial thioredoxin-like protein that can be translocated into host cells and mimic the endogenous TRX1 to target ASK1-MAPK signaling, finally facilitating bacterial pathogenesis. This work expands our understanding of bacterial thioredoxins in manipulating host innate immunity.

## Introduction

Thioredoxins (Trxs) are small redox-active molecules ubiquitously expressed in all taxa, from bacteria to mammals, containing a conserved redox catalytic CXXC (-Cys-X-X-Cys-)-motif that links the second β-strand to the second α-helix [1]. In mammalian cells, interactions between cytosolic TRX1 and apoptosis signal-regulating kinase 1 (ASK1) suppress the activation of c-Jun N-terminal kinase (JNK)-, Erk1/2-, and p38-MAPK signaling cascades in response to various stress stimuli and activate a number of transcription factors that regulate various aspects of cell growth and survival [2–8].

Mitogen-activated protein kinase (MAPK) signal transduction, which involves sequential activation and amplification of downstream kinases, is a high-value target of bacterial pathogens during infection process [9]. Bacteria have developed several strategies to target MAPK pathway in order to subvert their functions, one of which is that bacterial virulence factors operate as mimics of host proteins [10–11]. Anthrax toxin (lethal factor, LF), produced by the bacterium *Bacillus anthracis*, was proved directly inhibiting MAPKs by cleavage of the amino terminus of MAPKK1 and MAPKK2 [12]. Since then, YopJ from *Yersinia pestis* [13–14] and AvrA from *Salmonella* [15] was proved function as acetyl transferases that covalently modify key serine and threonine residues of MAPKs, regulating the transcription of pro-survival genes during infection. OspF from *Shigella flexneri*, which is homologous to the *Salmonella* SpvC and *Pseudomonas syringae* HopAI1, possesses the phosphor-threonine lyase activity, and irreversible dephosphorylate MAPKs by covalent modification and inhibit the inflammatory responses [16–17].

*Edwardsiella piscicida*, previously named *Edwardsiella tarda*, is an intracellular bacterium with broad cellular tropism, which is a pathogen primarily for fish [18–19]. Type III secretion system (T3SS) and type VI secretion system (T6SS) have been identified in this pathogen [20–23]. Moreover, *E*. *piscicida* EIB202 activates NLRC4 and NLRP3 inflammasomes via T3SS and inhibits the NLRP3 inflammasome via the T6SS effector EvpP [22]. Recently, we revealed that the wild-type *E*. *piscicida* (EIB202) replicates and induces pyroptosis in macrophages [24]. The macrophage-released *E*. *piscicida* population exhibits enhanced infectivity both *in vitro* and *in vivo* [24], and displays a reprogrammed transcriptional profile characterized by the upregulation of T3SS/T6SS-related genes as well as some uncharacterized genes [20–21, 23–24]. Thus, we hypothesize that the infection-induced *E*. *piscicida* genes might play important roles during infection. In this study, we found that one of the most significant infection-induced *E*. *piscicida* genes, *trxlp*, could be secreted and translocated into the cytosol of host cells upon infection, showing a conserved crystal structure to the host thioredoxin protein. Furthermore, Trxlp could mimic endogenous TRX1 to directly target the TRX-binding domain of ASK1 (ASK1-TBD) and inhibit its activation, subsequently suppressing Erk/p38-MAPK signaling, correlating to limit bacterial virulence and replication *in vivo*. Collectively, this study advances our understanding of bacterial thioredoxin as a virulence effector that mimics the host endogenous protein in manipulating innate immunity.

## Results

### Identification of Trxlp as a novel virulence effector

Based on the comparison of the global gene transcriptional profiles between macrophage-released and DMEM-cultured EIB202 [24], we annotated 10 most highly upregulated genes of EIB202 after macrophage infection (S1 Table). The gene of ETAE_2186 was among the top 3 upregulated genes and was annotated as a hypothetical thioredoxin (S1 Table); here, we named it Trx-like protein (Trxlp). Notably, during EIB202 infection in macrophages, *trxlp* was dramatically upregulated, while the other 10 Trx antioxidant family proteins in this strain were not significantly induced during infection (S1 Fig).

**Fig 1:**
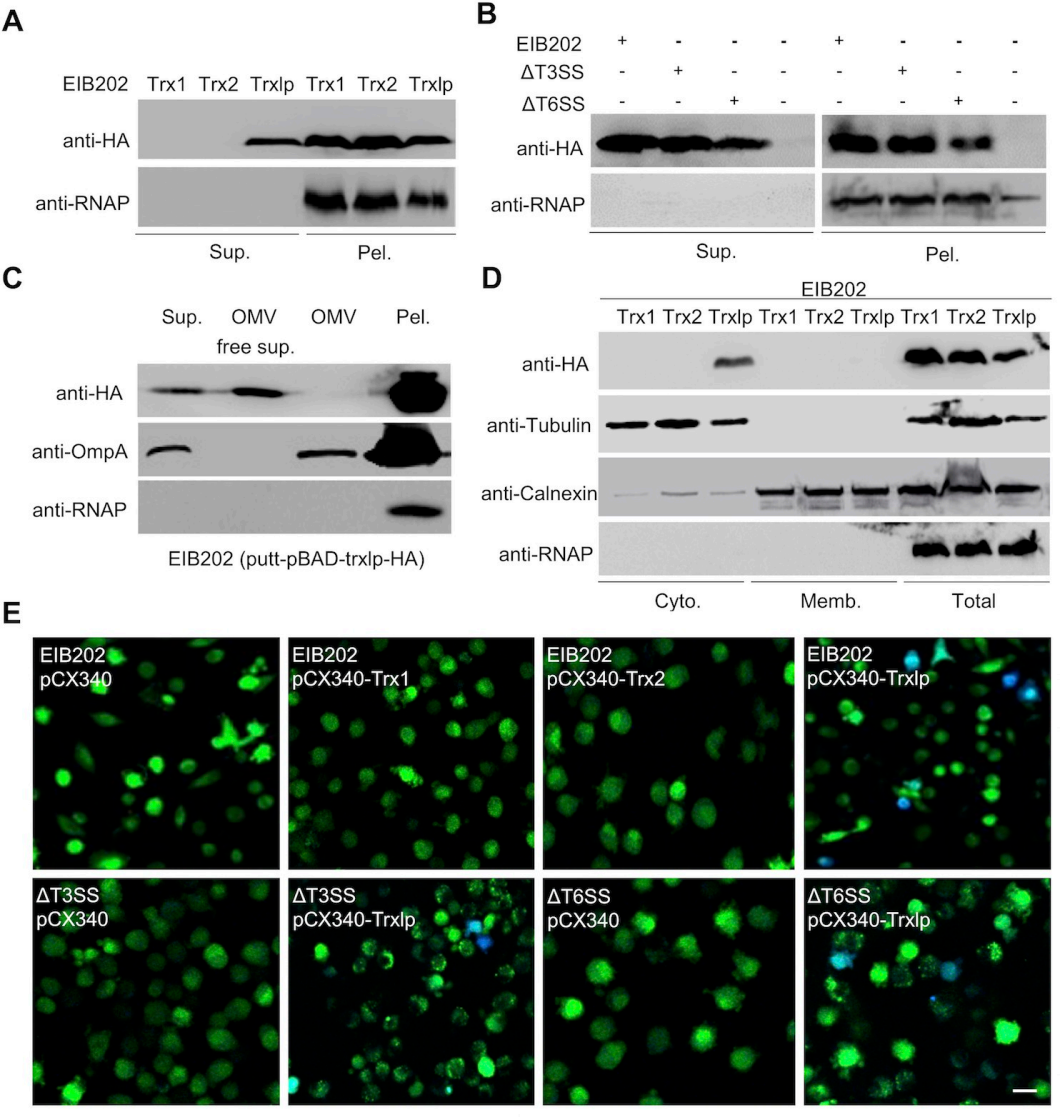
Identification of Trxlp as a novel virulence effector. **(A)** Supernatants (Sup.) and pellets (Pel.) from Trxs-HA fusion-expressing EIB202 were analyzed by immunoblotting. The bacteria were cultured in DMEM for 16 h and fractioned into pellets and supernatants. Anti-HA antibody was used to probe the Trxs-HA fusion protein, and anti-RNAP antibody was used as a bacterial cytosolic marker. **(B)** Supernatants (Sup.) and pellets (Pel.) from Trxlp-HA fusion-expressing EIB202, ΔT3SS, ΔT6SS *E*. *piscicida*, *or* HA-expression EIB202 were analyzed by immunoblotting. The bacteria were cultured in DMEM for 16 h and divided into pellets and supernatants. Anti-HA was used for Trxs-HA fusion protein probing, and anti-RNAP was used as a bacterial cytosolic marker. **(C)** Supernatants (Sup.) with/without Outer membrane vesicles (OMVs), OMVs, and pellets (Pel.) from Trxlp-HA fusion-expressing EIB202 were analyzed by immunoblotting. Anti-HA and anti-OmpA were used to probe Trxlp-HA fusion protein outer membrane protein vesicles, respectively, and anti-RNAP was used as a bacterial cytosolic marker. **(D)** Assays of intracellular translocation of Trxlp by immunoblotting. HeLa cells were infected with Trxs-HA fusion-expressing EIB202 at a MOI of 100 for 2 hours; cells were subjected to differential centrifugation to separate subcellular fractions. These fractions were analyzed by immunoblotting, as indicated. Calnexin, a marker of the host cell membrane; β-tubulin, a marker of cytosolic proteins; RNAP, a bacterial cytosolic protein. (**E**) Assay of intracellular translocation of indicated Trxs by fusing with β-lactamase. HeLa cells were infected with EIB202, ΔT3SS or ΔT6SS *E*. *piscicida* expressing effector-TEM fusion protein at a MOI of 100. Eight hours after infection, cells were loaded with CCF4-AM. Translocation of effector-TEM into the cell cytosol results in the cleavage of CCF4-AM, causing the emission of blue fluorescence. Uncleaved CCF4-AM emits green fluorescence. Scale bar = 50 μm. TEM, TEM-1-β-lactamase. **(A-E)** Data are representative of at least 3 experiments.

*E*. *piscicida* possesses multiple secretion systems to deliver virulence factors during infection [20–23, 25]. To investigate whether Trxlp is a secreted protein, we assessed the production of Trxlp-HA in EIB202 strains grown in DMEM. The robust secretion of Trxlp, but not classical Trx1 or Trx2, was detected in the bacterial supernatants (Fig 1A). Importantly, comparable production of Trx1, Trx2, and Trxlp-HA was observed in the pellets of EIB202 (Fig 1A). In addition, Trxlp was secreted in T3SS-deficient or T6SS-deficient EIB202 at a comparable level to that in wild-type strains (Fig 1B). Moreover, Trxlp was not detected in the fraction of the outer membrane vesicles (OMVs) (Fig 1C), which were reported to be vehicles delivering bioactive proteins, toxins, and virulence factors [26,27]. Thus, these results indicate that the Trxlp secretion was independent of T3SS, T6SS, or OMV pathways.

We next investigated the intracellular localization of Trxlp during infection. The subcellular fractionation of HeLa cells infected with EIB202 revealed that Trxlp is translocated into HeLa cells and localized in the cytosolic fraction, but not in the membrane fraction (Fig 1D). Moreover, immunofluorescence microscopy revealed that Trxlp, but not Trx1 and Trx2, localized into the cytosol of HeLa cells (S2 Fig). Furthermore, infection of HeLa cells revealed that Trxlp, but not Trx1 or Trx2, could be translocated into host cells through the β-lactamase reporter system, and the translocation of Trxlp was independent of T3SS or T6SS (Fig 1E). Thus, our results suggest that Trxlp is a novel *E*. *piscicida* virulence effector that can be translocated into the cytosol of host cells during infection.

### Crystallization and structure analysis of Trxlp

**Fig 2:**
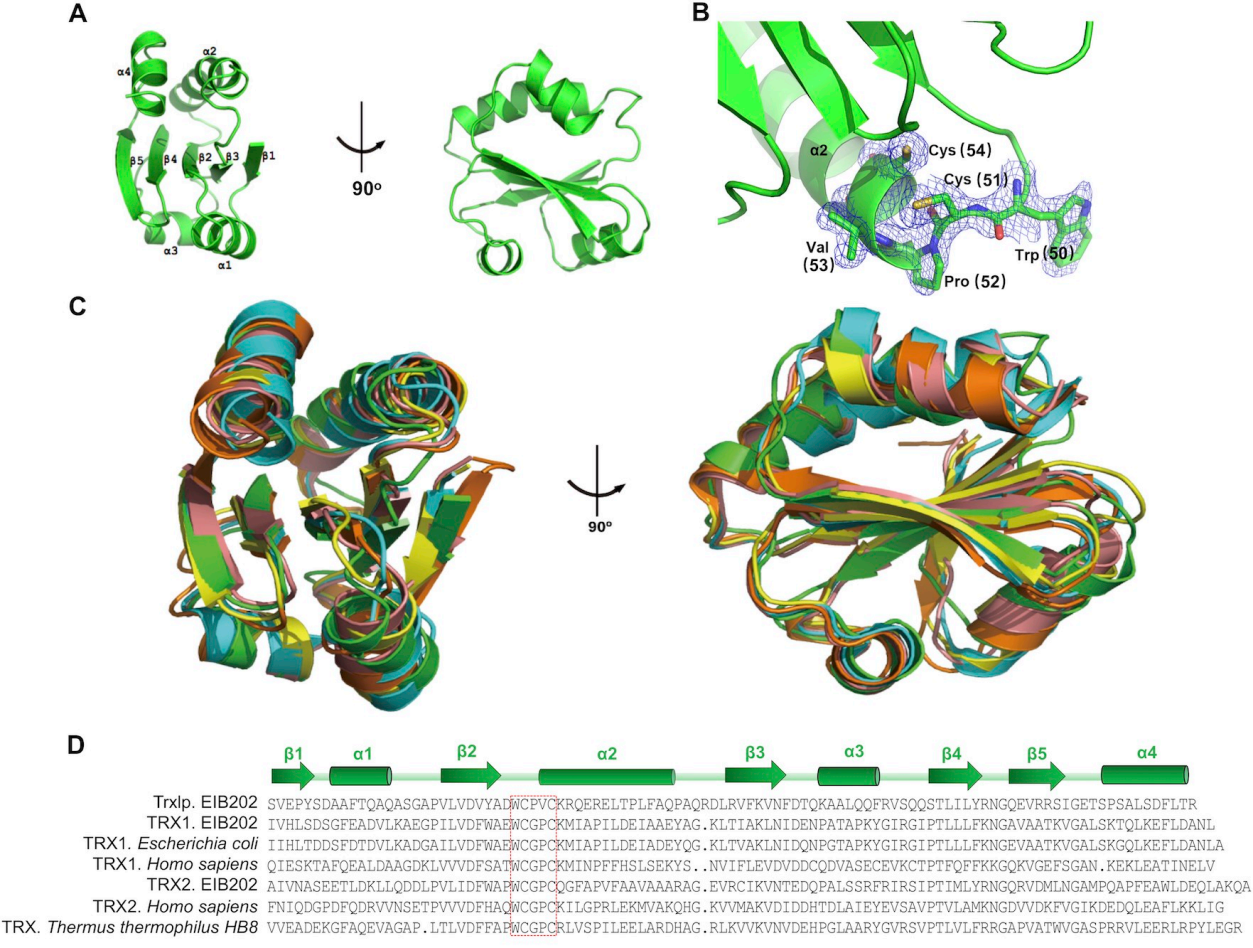
Crystal structure analysis of Trxlp. **(A)** Two orthogonal views of the overall structure of Trxlp. **(B)** Trxlp is crystallized in the reduced form. The electron density (2Fo-Fc) map, contoured at 1δ, is shown for the CXXC motif. **(C)**Superimposition of thioredoxin from various species. Trxlp (PDB: 5ZF2), green; human thioredoxin 1 (PDB: 1ERT), yellow; human thioredoxin 2 (PDB: 1UVZ), cyan; *Escherichia coli* thioredoxin 1 (PDB:2TRX), orange; *Thermus thermophilus* thioredoxin 1 (PDB: 2YZU), red. **(D)** The multiple sequence alignments of thioredoxin antioxidant system-related amino acid sequence with other known Trx sequences. The sequences used to generate the multiple sequence alignments are as follows: EIB202 Trxlp and TRX1 (GenBank accession no. ACY85021.1, ACY82946.1), *E*. *coli* TRX1 (GenBank accession no. AFG42725.1), *Homo sapiens* TRX1 (GenBank accession no. AFH41799.1), EIB202 TRX2 (GenBank accession no. ACY83406.1), *Homo sapiens* TRX2 (GenBank accession no. AAF86467.1), and *T*. *thermophilus* HB8 TRX (GenBank accession no. BAD71304.1). Residues that form the conserved redox catalytic motif are indicated in the red box. Secondary structure assignments based on Trxlp structure are shown as cylinders (α-helices) and arrows (β-strands).

To better characterization the importance of this virulence effector, we solved the crystal structure of Trxlp by molecular replacement using *Thermus thermophilus* Trx (PDB:2YZU) as the initial model. The structure was refined to an R-value of 17.7% (*R*_free_ = 21.5%) with good geometry at a resolution of 1.98 Å (S2 Table). Trxlp displays a similar structure to that of the canonical Trx fold, consisting of a β1-α1-β2-α2-β3-α3-β4-β5-α4 topology (Fig 2A). The structure obtained here is in reduced form, as evidence by the 3.3 Å distance between 2 sulfur atoms of 2 cysteines in the CXXC motif (Fig 2B). Trxlp contains a central β-sheet composed of 3 parallel (β1, β2, β3) and 2 antiparallel strands (β4, β5). This central β-sheet is sandwiched by 2 layers of helices: a bottom layer with 2 helices (α1, α3) and a top layer with the other 2 helices (α2, α4). Trxlp can be superimposed to Trxs from other species, with root-main-square deviation (rmsd) values ranging from 1.54 to 1.74 Å for around 100 equivalent C_α_ pairs (Figs 2C and S3). Minor conformational differences among these structures were observed in flexible loop regions connecting α helices and β strands. Sequence-based homology searches and motif and PHYRE2 fold recognition analyses revealed that Trxlp contains a CXXC motif comparable to that of classical Trxs in other bacteria (Fig 2B and 2D). Unlike Trx2 identified in EIB202, both Trx1 and Trxlp contain only one CXXC-motif site and lack a mitochondrial-targeting sequence (S4A Fig). Collectively, Trxlp was identified as a Trx family protein with a conserved redox catalytic CXXC motif.

Thioredoxin is a ubiquitous thiol oxidoreductase that regulates the cellular redox status [7, 8]. Thus, we analyzed the enzymatic function of Trxlp. Consistent with the number of CXXC motifs, the incubation of purified Trx2 resulted in a further reduction in the artificial disulfide substrate DTNB [28] compared to incubation with purified Trx1 or Trxlp (S4B Fig). Moreover, the reduction of insulin by dithiothreitol (DTT) [29, 30] at pH 7.0 was assessed in the absence or presence of EIB202 Trx1, Trx2, and Trxlp. Notably, both Trx1 and Trx2 catalyzed the reduction of insulin by DTT, as quantified by the onset of aggregation, while Trxlp was much less active than the classical reducing Trxs in EIB202 (S4C Fig). Taken together, unlike the classical Trxs, Trxlp might not exhibit robust redox activity. Thus, this novel Trx family protein and its role during *E*. *piscicida* infection should be characterized further.

### Trxlp interacts with ASK1-TBD residues to abrogate ASK1 phosphorylation

**Fig 3:**
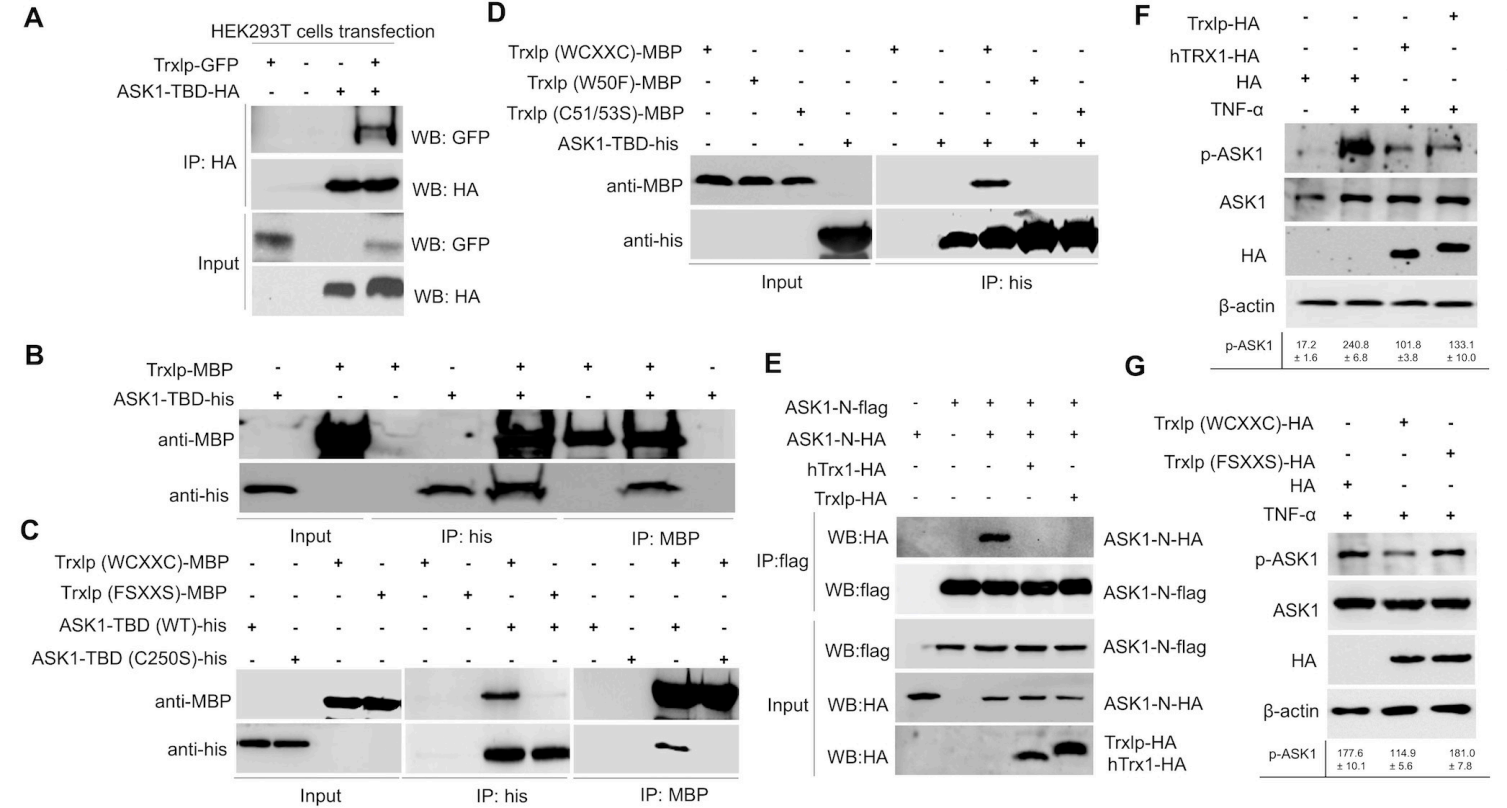
Trxlp interacts with ASK1-TBD residues to abrogate ASK1 activation. **(A)** Immunoprecipitation assay of Trxlp and ASK1-TBD. HEK293T cells were cotransfected with indicated Trxlp-GFP and ASK1-TBD-HA expression vectors and cultured for 36 h. The cells were then lysed in lysis buffer and immunoprecipitated with HA beads for 4 h. Anti-HA and anti-GFP antibodies were used for immunoblotting analysis. (**B**-**D)** Pulldown assays of interaction between purified ASK1-TBD-His and Trxlp-MBP. Immunoblotting to detect ASK1-TBD-His and Trxlp-MBP is shown. **(E)** Trxlp and human Trx1 inhibit homophilic interaction of ASK1 through N-terminal region. HEK293T cells were transfected with the indicated combinations of ASK1-N-HA, ASK1-N-flag, Trxlp-HA and human TRX1-HA plasmids. Cell lysates were immunoprecipitated with anti-flag antibody. Immunoprecipitates and aliquots of each lysate were subjected to SDS-PAGE followed by immunoblotting with indicated antibodies. (**F** and **G)** Trxlp inhibits the phosphorylation of endogenous ASK1. HEK293T cells transfected with Trxlp-HA, human TRX1-HA or mutant Trxlp (FSXXS), respectively, and pretreated with 100 ng/ml TNF-α for 30 min after 36 h. The phosphorylation of endogenous ASK1 and ASK1 in 293T cells was detected by immunoblotting using anti-phospho-ASK1 (Thr845) and anti-ASK1 antibodies. Immunoblotting results for HA and β-actin are also shown. (**A-G)** The signal intensities were quantitatively analyzed using Quantity one software. Data (Means ± SD) are representative of at least 3 experiments.

TRX1, an endogenous ubiquitous oxidoreductase, is a physiological inhibitor of ASK1 via interactions with its N-terminal region, termed the Trx-binding domain in human cells (ASK1-TBD) [4, 31]. Trxlp shares a conserved structure and critical WCXXC-motif site not only with bacterial Trxs, but also with mammalian Trxs (Figs 2C, 2D and S3). Thus, we hypothesize that Trxlp can mimic endogenous TRX1 when it is translocated into the cytosol of host cells during infection. To determine whether Trxlp can interact with ASK1-TBD, we analyzed the immunoprecipitation of ASK1-TBD followed by immunoblotting to detect Trxlp, which showed that ASK1 associates with Trxlp via the Trx-binding domain (Fig 3A). Moreover, an *in vitro* pulldown assay also revealed an association between Trxlp and ASK1-TBD (Fig 3B). Notably, either mutation in Trxlp (W50F, C51/53S, FSXXS) or mutation in ASK1-TBD (C250S) significantly impaired the interactions between them (Fig 3C and 3D). Thus, consistent with previous results regarding endogenous TRX1 binding with ASK1[4, 31], we identified, for the first time, a bacterial Trx family protein that can bind to ASK1-TBD via the conserved redox catalytic WCXXC motif.

Since the N-terminal Trx-binding domain of ASK1 is necessary and sufficient for its association with Trx, which inhibits ASK1 activity by disrupting N-terminal coiled-coil (NCC) domain homophilic interactions [32], it is interesting to test whether Trxlp could inhibit the homophilic interaction via the NCC domain of ASK1 (ASK1-N). Thus, we examined the effect of Trxlp and human TRX1 on the homophilic interaction of ASK1-N by cotransfection analysis. The association of ASK1-N-HA with ASK1-N-Flag was inhibited by both human TRX1 and Trxlp *in vitro* (Fig 3E). These findings suggest that the NCC domain-mediated homophilic interaction of ASK1 is suppressed by the association with Trxlp *in vitro*.

The phosphorylation of the conserved threonine residue at the activation loop is essential for the kinase activity of human ASK1 [33]. In this study, we further analyzed the effects of Trxlp on the kinase activity of ASK1. We detected robust phosphorylation of ASK1 in the presence of TNF-α, but this phosphorylation was significantly reduced in the presence of wild-type Trxlp or human TRX1 as a control (Fig 3F). However, the mutant Trxlp (FSXXS) did not abrogate the TNF-α-induced ASK1 phosphorylation (Fig 3G). Taken together, our results reveal that Trxlp can mimic endogenous TRX1 to inhibit the phosphorylation of ASK1.

### Trxlp limits ASK1-Erk/p38-MAPKs activation during *E*. *piscicida* infection

**Fig 4:**
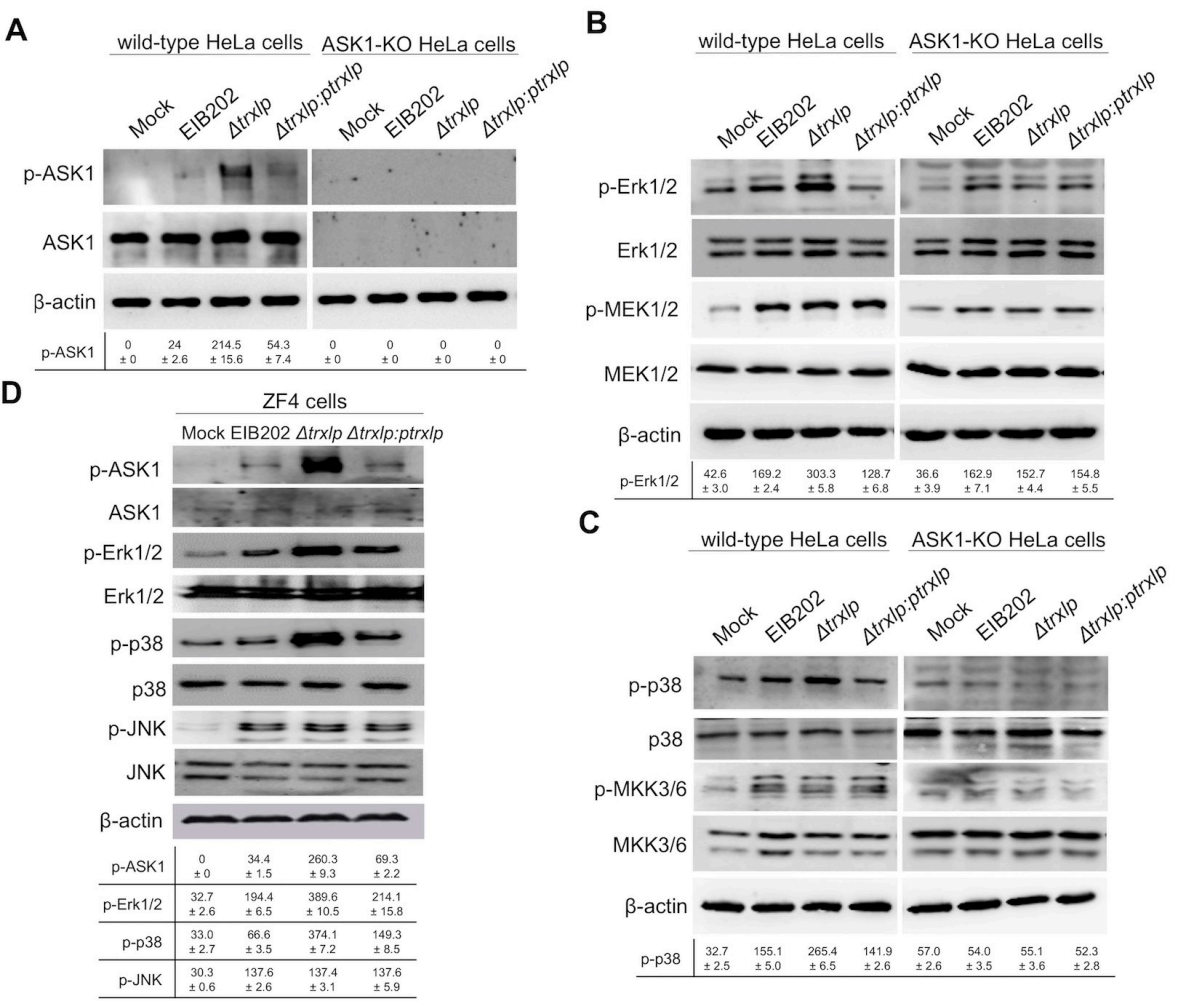
Trxlp suppresses the phosphorylation of Erk1/2 and p38 dependent on ASK1 during *E*. *piscicida* infection. (**A-C)** Immunoblotting assays of ASK1, MAP2Ks and MAPKs activation during *E*. *piscicida* infection in HeLa cells. Wild-type HeLa cells and ASK1-KO HeLa cells were infected with EIB202, *Δtrxlp*, or *trxlp-*complemented *E*. *piscicida* at a MOI of 100 for 2 h. Cell lysates were collected and probed for anti-phospho-ASK1 and anti-ASK1 antibodies (A), or probed for anti-phospho-Erk1/2 and anti-Erk1/2, and anti-phospho-MEK1/2 and MEK1/2 antibodies (B), or probed for anti-phospho-p38α and anti-p38α, and anti-phospho-MKK3/6 and MKK3/6 antibodies (C). **(D)** Immunoblotting assays of ASK1 and MAPKs activation during *E*. *piscicida* infection in Zebrafish fibroblasts (ZF4). ZF4 cells were infected with EIB202, *Δtrxlp*, or *trxlp-*complemented *E*. *piscicida* for 2 h at a MOI of 10. Cell lysates were probed with anti-phospho-ASK1 and anti-ASK1, anti-phospho-Erk1/2 and anti-Erk1/2, anti-phospho-p38 and anti-p38, anti-phospho-JNK and anti-JNK antibodies. **(A-D)** β-Actin is shown as a loading control. The signal intensities were quantitatively analyzed using Quantity one software. Data (Means ± SD) are representative of at least 3 experiments.

During HeLa cells infection, Δ*trxlp* triggered robust ASK1 phosphorylation compared to EIB202, and Trxlp complementation neutralized ASK1 phosphorylation, suggesting that Trxlp critically participates in regulating ASK1 activation (Fig 4A). Since ASK1 is an upstream signaling partner of the MAPKKK family and its phosphorylation induces the activation of the MAPK signaling cascade in response to various stress stimuli [3–5, 34], it is interesting to examine whether Trxlp regulates the activation of the MAPKs or NF-κB pathways during *E*. *piscicida* infection. Interestingly, significantly increased phosphorylation levels of Erk1/2 and p38 were induced in HeLa cells infected by Δ*trxlp* than by the isogenic wild-type strain, or Δ*trx1* and Δ*trx2* (S5A Fig); however, no detectable difference in JNK phosphorylation and IκBα degradation was observed between them (S5A Fig). In addition, we demonstrated that the phosphorylation of the Erk1/2- and p38 -MAPK pathways was induced by TNF-α in untransfected HEK293T cells, but it was dramatically reduced in cells expressing either human TRX1 or Trxlp (S5B Fig); meanwhile, the suppression of Erk1/2 and p38 phosphorylation was abrogated in cells expressing the mutant Trxlp (FSXXS) (S5C Fig). Thus, these results suggest that Trxlp can suppress the phosphorylation of Erk1/2- and p38-MAPK signaling via the conserved redox catalytic WCXXC motif.

To further validate the role of ASK1 in *E*. *piscicida*-induced MAPK pathway activation, the CRISPR/Cas9 genome-editing tool was applied to knockout (KO) ASK1 in HeLa cells using *ask1-*specific guide RNA. The Trxlp-suppressed Erk-1/2 activation effect was abrogated in ASK1-KO HeLa cells, while the MEK1/2 activation was not affected during *E*. *piscicida* strains infection (Fig 4B). Moreover, the Trxlp-suppressed p38α activation effect was also abrogated in ASK1-KO HeLa cells, but the activation of MKK3/6 was not regulated by Trxlp (Fig 4C). Simultaneously, consistent with above results, the activation of JNK, or its upstream kinase MKK7 was also not regulated by Trxlp (S5D Fig). Collectively, these results suggest that Trxlp could inhibit the activation of ASK1 and thus limit Erk1/2- and p38-MAPK pathways through a MAPKK-independent mechanism during infection.

Given that *E*. *piscicida* is a broad-range intracellular pathogen affecting from fish to mammals [18], we subsequently investigated the role of Trxlp using a zebrafish fibroblasts (ZF4). Upon infection, ASK1 phosphorylation was enhanced in cells infected with Δ*trxlp* compared to the isogenic wild-type strain, but it was reduced to normal level when ZF4 cells were infected with a *trxlp-*complemented strain (Fig 4D). Consistent with the results obtained using mammalian cells, Erk1/2 and p38 phosphorylation levels were also enhanced in ZF4 cells infected with Δ*trxlp*, but not in cells infected with *trxlp-*complemented strain (Fig 4D). Furthermore, comparable phosphorylation levels of JNK and MAP2Ks, including MEK1/2, MKK3/6, MKK4, and MKK7, were observed between wild-type and Δ*trxlp* infected ZF4 cells (S6A Fig).

It is known that p38-MAPK or Erk1/2-MAPK potently control the production of many pro- or anti-inflammatory cytokines, which are critical for host immunity [9, 35, 36]. Thus, we further analyzed the regulation of cytokine production during *E*. *piscicida* infection. *Δtrxlp* induced the greatly increased transcription of TNF-α and IL-10 than wild type strain in ZF4 cells, which was counteracted by Trxlp complementation (S6B and S6C Fig). Simultaneously, ELISA assay demonstrated that the production of TNF-α was significantly induced in cells infected with the Trxlp mutant strain (S6G Fig). However, the transcription levels of *IL-6*, *cxcl8*, and *IFN-γ* were not regulated by Trxlp during *E*. *piscicida* infection (S6D to S6F Fig). Taken together, these observations demonstrate a key role of Trxlp in suppression of ASK1-Erk/p38-MAPK signaling and thereby regulation of inflammatory cytokines expression during *E*. *piscicida* infection.

### Trxlp promotes bacterial colonization *in vivo*

**Fig 5:**
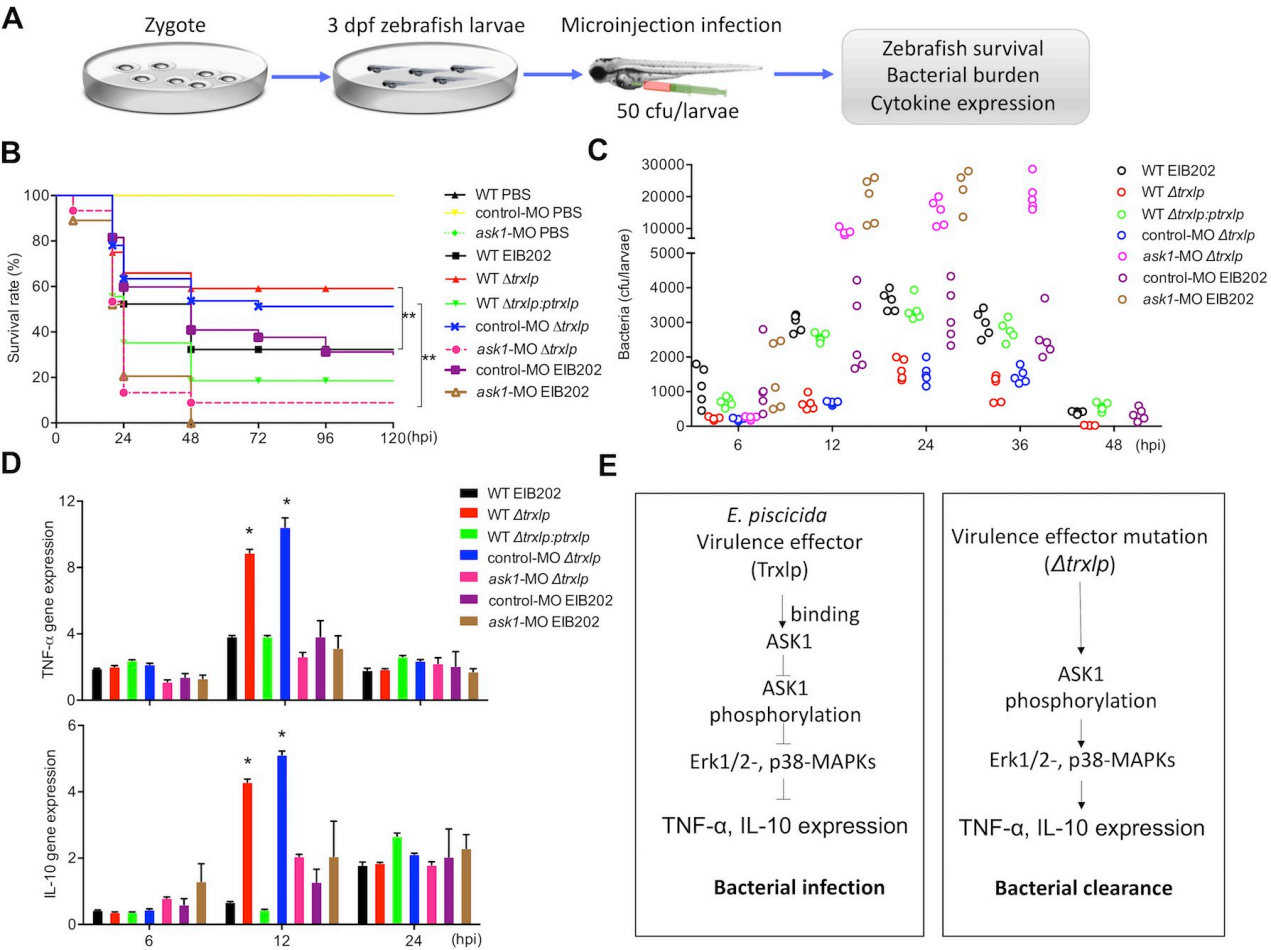
Trxlp promotes bacterial colonization *in vivo*. **(A)** Schematic representation of the zebrafish larvae microinjection infection model. Zebrafish larvae were maintained in E3 medium for up to 8 days post-fertilization (dpf). Bacteria were microinjected into the yolk sac of zebrafish larvae at 3 dpf, and the survival, bacterial burden, and cytokine expression were analyzed. **(B)** Wild-type (WT) or *ask1*-MO zebrafish larvae were infected with EIB202, *Δtrxlp*, *trxlp-*complemented *E*. *piscicida* (50 CFUs/larvae), or PBS as a control, respectively. The survival of zebrafish larvae was monitored for 5 days. *n* = 50 fish per group. Data shown are from 3 representative experiments. ** *p* < 0.01. **(C)** The zebrafish larvae were infected as in Fig 5B and collected at the indicated post-infection time points, and homogenates were plated to determine the bacterial CFUs per larvae. *n* = 5 fish per group at each time point. Data are representative of at least 3 experiments. (**D)** The zebrafish larvae were infected as in Fig 5B, and mRNA levels of *TNF-α* and *IL-10* in indicated zebrafish larvae infected with EIB202, *Δtrxlp*, or *trxlp-*complemented *E*. *piscicida* at indicated time points were determined by qRT-PCR. PBS-treated zebrafish was used as the control. Data (means ± SD) are representation of 3 experiments. * *p* < 0.05. **(E)** Diagram of Trxlp function during *E*. *piscicida* infection process. The novel virulence effector Trxlp targets and inhibits the phosphorylation of ASK1, thereby suppressing Erk1/2- and p38-MAPKs and inhibiting the expression of TNF-α and IL-10 during infection.

Given the similarity between zebrafish MAPK pathways and those of mammals [37, 38], to evaluate the function of Trxlp in the regulation of ASK1-MAPK signaling *in vivo*, we developed a microinjection infection model [38] using 3 days post fertilized (dpf) zebrafish larvae for analyzing the pathogenesis of *E*. *piscicida* (Fig 5A). We found that zebrafish larvae were more susceptible to EIB202 or *trxlp-*complemented strain than to Δ*trxlp* during infection (Fig 5B), consistent with the reduced pathogen loads in Δ*trxlp*-infected zebrafish larvae (Fig 5C). In addition, we found that infection with EIB202 induced the expression of *TNF-α* and *IL-10* transcripts, which was further enhanced in zebrafish larvae infected with Δ*trxlp* (Fig 5D). The enhancement of cytokine expression observed with Δ*trxlp* was abrogated when zebrafish larvae were infected with the *trxlp-*complemented strain (Fig 5D). However, the transcript levels of *IL-6*, *cxcl8*, and *IFN-γ* were not regulated by Trxlp during *E*. *piscicida* infection (S7B to S7D Fig). These results indicate that the novel virulence effector Trxlp facilitates bacterial survival and virulence *in vivo*.

To analyze the involvement of ASK1 in promoting immune defense against *E*. *piscicida in vivo*, the morpholino (MO) oligonucleotide was designed to block *ask1* translation and injected into embryos at the one-cell stage. Immunoblotting showed that this MO effectively knocked down *ask1* for up to 7 dpf (S7A Fig). Following infection with 50 CFUs of EIB202 or Δ*trxlp*, *ask1*-MO larvae succumbed more rapidly than the control-MO larvae (Fig 5B). Consistent with a role of ASK1 in maintaining homeostasis, *ask1*-MO larvae had significantly higher pathogen loads during either EIB202 or Δ*trxlp* infection than in control-MO larvae (Fig 5C and S3 Table). Furthermore, *ask1*-MO larvae exhibited significantly reduced *TNF-α* and *IL-10* transcript levels during either EIB202 or Δ*trxlp* infection (Fig 5D), while the transcript levels of *IL-6*, *cxcl8*, and *IFN-γ* were not affected compared with infected control-MO larvae (S7B to S7D Fig). Taken together, our results suggest that the ASK1 might be a host target of the *E*. *piscicida* virulence effector Trxlp during infection *in vivo*, and the activation of ASK1-MAPK signaling cascades plays critical role in innate immunity (Fig 5E).

## Discussion

Trx was originally considered an important conserved family for protection against ROS by reducing peroxides to harmless products [7–8]. The antioxidant defense system of microorganisms comprises various conserved antioxidant molecules [8], but little is known about the specific roles of these molecules during infection. In this study, we analyzed all annotated Trx family proteins of *E*. *piscicida*, and found that only Trxlp was significantly upregulated when compared with the levels of classical bacterial Trx family proteins during infection (Figs 1A and S1). However, this Trxlp showed significantly lower redox activity than the classical reducing Trxs in EIB202 (S3 Fig). This is the first report that a unique bacterial thioredoxin was utilized as a virulence effector to interfere with host antibacterial signaling, which expands our understanding of the bacterial Trx family proteins that not only catalyze protein disulfide reductase, but also function as virulence effectors during infection.

In mammals, endogenous Trxs maintain the cellular redox state and regulate cell proliferation by acting as electron donors of ribonucleotide reductase [7–8]. Previous studies have shown that endogenous TRX1 is a negative regulator of ASK1 and constantly forms an inactive complex with ASK1 by associating with the N-terminal regulatory domain and inhibiting homophilic interactions with ASK1 [4, 31,32]. Moreover, the ring finger domains of TRAF2 and TRAF6 downstream are required to accelerate the N-terminal homophilic interaction of ASK1 [32], and the deregulation of ASK1 affects cell fate, such as survival and apoptosis, which is required for mammalian innate immunity [3, 39]. Interestingly, we identified a bacterial virulence effector, Trxlp, which could mimic endogenous TRX1 to bind with the ASK1 and block its homophilic interactions, however, the precise mechanism of bacterial Trxs in manipulating host innate immune signaling during infection remains an open question.

Previous studies have shown that ASK1 is activated in response to a variety of stress-related stimuli via distinct mechanisms and activates MKK4 and MKK3, which in turn activate JNK and p38 [3]. In our study, either in mammalian cells or fish cells infected with *E*. *piscicida*, Trxlp could regulate the phosphorylation of ASK1. However, quite different from previous observations, we found that the bacterial infection-engaged inhibition of ASK1 was responsible for regulating Erk1/2- and p38-MAPKs activation, but not JNK-MAPK signaling (Figs 4 and S5). Although, this was consistent with a previous study that observed the down-regulation of Trx induced ROS-mediated ASK1-Erk/p38-MAPK activation in human promonocyte cells during Japanese encephalitis virus infection [5], we still expecting that multiple effectors might alter the JNK-MAPK pathway adversely during infection. Besides, another interesting issue is the activation of MAP2K pathways were not affected during *E*. *piscicida* infection, which was quite different from the classical activation of ASK1-MAP2K-MAPK signaling cascades under stress-related stimulus [3]. This discrepancy suggests a possibility that a MAP2K signaling independent pathway might be triggered by ASK1 to activate p38- and Erk1/2-MAPKs during infection.

**Fig 6:**
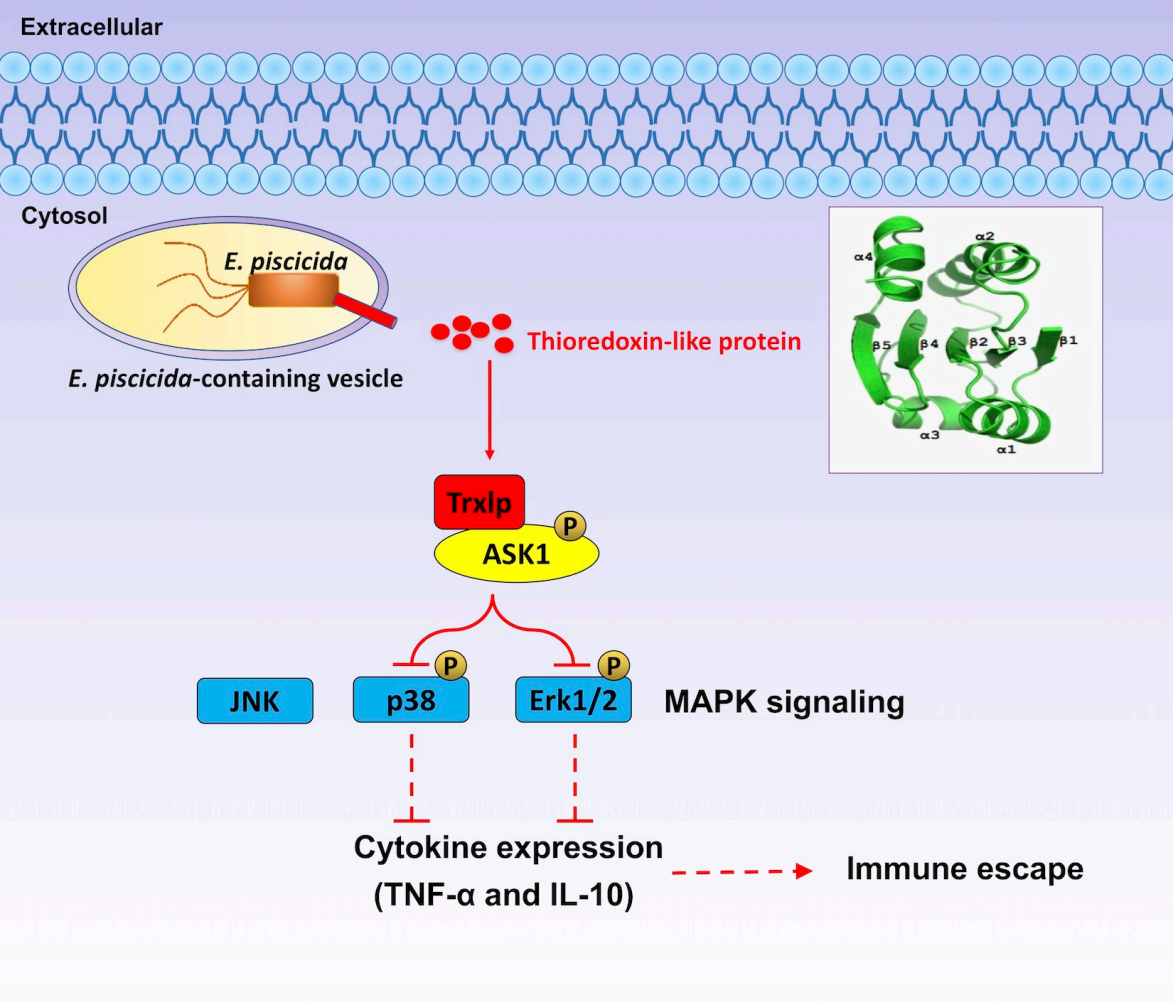
Proposed mechanism for *E*. *piscicida* thioredoxin-like protein. The novel bacterial virulence effector, thioredoxin-like protein (Trxlp), could be translocated into cytosol of host cells, mimicking the endogenous TRX1 to target ASK1-MAPK signaling to restrict the innate immune response *in vivo*.

In *E*. *piscicida*, multiple effectors, especially those using T3SS and T6SS systems, cooperate or feedback with each other to directly mimic, intercept, or modify the function of key host factors engaged in a wide range of cellular processes, including innate immune signaling, cytoskeletal dynamics, membrane trafficking, phosphoinositide lipid metabolism, and cell signaling, which finally influence bacterial dissemination and survival [24]. EseH inhibited phosphorylation of Erk1/2, p38α and JNK MAPK pathways in host cells, but had no effect on the NF-kB pathway [40]. EvpP significantly suppressed JNK activation, thus impairing oligomerization of the inflammasome adaptor ASC [22]. EseK also can inhibit MAPK phosphorylation and promotes bacterial colonization in zebrafish larvae [41]. Here, we present a comprehensive functional interpretation for the newly-identified virulence effector mimicking host Trx to regulate host ASK1-Erk/p38 MAPKs axis and promote bacterial infection *in vivo* (Fig 6). However, many details upstream of its intracellular behaviors, including how the bacterium responds to infectious signals to upregulate Trxlp transcription and how the secretion and translocation of Trxlp are coordinated during infection remain unknown. Thus, supplementary with our previous identified effectors in regulating MAPK pathways, our study clarified the roles of Trxlp in inhibited Erk1/2, p38α-MAPK pathways in host cells, and the dynamic or redundant roles of these effectors in regulating MAPK signaling during *E*. *piscicida* infection to achieve its infectious goal remains to be clarified.

Taken together, our results provide the first comprehensive, functional analysis of the *E*. *piscicida* Trx family protein Trxlp, a novel virulence effector that mimics endogenous TRX1 to target ASK1 and suppress its activation, thereby inhibiting the phosphorylation of Erk1/2- and p38-MAPKs and disrupting the expression of inflammatory cytokines and diminishing the host antibacterial defense. These findings provide insight into the mechanisms underlying the regulation of ASK1, suggest that the development of drugs targeting ASK1 may be useful for the treatment of bacterial infectious diseases, and advance our knowledge of the general biology of pathogen–host interactions.

## Materials and methods

### Ethics statement

Animal experiments were conducted according to the Guide for the Care and Use of Medical Laboratory Animals (Ministry of Health, People’s Republic of China) and ethically approved by The Laboratory Animal Ethical Committee of East China University of Science and Technology (Protocol #2006272). All the infection experiments were conducted as a completely randomized design, and the analyses were performed in a blinded manner.

### Culture conditions

The bacterial strains used in this study are described in S4 Table. Wild-type *E*. *piscicida* EIB202 (CCTCC M208068) and indicated mutants were grown in tryptic soy broth (TSB; BD Biosciences), tryptic soy agar (TSA) or Dulbecco’s modified eagle medium (DMEM; Invitrogen) at 30°C. *Escherichia coli* was cultured in Luria-Bertani (LB; BD Biosciences) broth or agar at 37°C. Antibiotics were added to the media at the following concentrations: ampicillin (Amp), 100 μg/ml; kanamycin (Km), 50 μg/ml; colistin (Col), 16.7 μg/ml.

HEK293T (ATCC CRL-11268), J774A.1 (ATCC TIB-67) and HeLa cells (ATCC CCL-2) were grown at 37°C in DMEM supplemented with 10% FBS and under a 5% (vol/vol) CO_2_ atmosphere. ZF4 cells (ATCC CRL-2050), established from 1-day-old zebrafish embryos, were grown at 28°C in DMEM/F12 medium supplemented with 10% FBS and under a 5% (vol/vol) CO_2_ atmosphere.

### Cloning, overexpression and mutagenesis

To detect the secretion of Trxs protein, wild-type, ΔT3SS and ΔT6SS *E*. *piscicida* expressing Trxs-HA protein were constructed, respectively. The Trx1, Trx2 and Trxlp sequences were amplified from *E*. *piscicida* genome and the constructed plasmids containing arabinose operon pUTt-pBAD-trx1, trx2, trxlp-HA were electroporated into indicated strains [42].

To construct the plasmids expressing Trxlp, hTRX1, N-terminal fragments of human ASK1-thioredoxin binding domain (ASK1-TBD, DNA encoding residues 88–302) and N-terminal coiled-coil domain of ASK1 (ASK1-N, DNA encoding residues 400) in eukaryotic cells, the plasmid pCDH-CMV-MCS-EF1-Puro (CD510B-1) was linearized with Xba I and EcoR I, and then the PCR products of genes containing compatible ends were inserted into the linearized pCDH plasmid using one step cloning kit (Vanzyme).

To construct the Δ*trx1*, Δ*trx2 and* Δ*trxlp E*. *piscicida*, an in-frame deletion mutation of *trxs* was generated by *sacB*-based allelic exchange as described [43, 44]. For example, the upstream and downstream fragments of *trxs* were fused by overlapping PCR. Primer pairs deletion-*trxs*-P1 plus deletion-*trxs*-P2 and deletion-*trxs*-P3 plus deletion-*trxs*-P4 were used. The resulting products were a 446-bp fragment containing the upstream region of *trx1* and a 435-bp fragment containing the downstream region of *trx1*. The resulting products were a 451-bp fragment containing the upstream region of *trx2* and a 437-bp fragment containing the downstream region of *trx2*. The resulting products were a 536-bp fragment containing the upstream region of *trxlp* and a 497-bp fragment containing the downstream region of *trxlp*. The fragments were cloned into the *sacB* suicide vector pDMK, linearized with Bgl II and Sph I, and the correct plasmids were introduced into *E*. *coli* CC118 λ*pir*. Single-crossover mutants were obtained by conjugal transfer of the resulting plasmid into wild-type *E*. *piscicida* (EIB202). Deletion mutants were screened on 10% sucrose-tryptic soy agar (TSA) plates. All the mutants were confirmed by PCR amplification of the respective DNA loci, and subsequent DNA sequencing of each PCR product.

ASK1-TBD and Trxlp were ligated into pET28a using the Nco I and Xho I sites, and the ASK1-TBD contains a sequence of 6 × His-tag at the C-terminal site, while the Trxlp contains a myelin basic protein (MBP) tag. The site-directed mutagenesis of ASK1-TBD or Trxlp was introduced using normal sequencing primers adjacent to multiple clone sites of plasmid as flanking primers. The recombinant plasmids containing genes were transformed into the *E*. *coli* strain BL21 (DE3), and the expressing proteins were water soluble after the induction of IPTG. All primers used for the construction of mutants are listed in S5 Table.

### Purification of Trxlp for crystallization

The DNA fragment containing the ETAE_2186 gene, amplified by PCR from the vector pET28a-his-ETAE_2186 (forward primer: 5’-GCGCGGATCCGTAGAGCCG GCCCTATAGCGACG-3’, reverse primer: 5’-GCGCCTCGAGTTAGCGGGTCAGA AAGTCAG-3’) was inserted into the pET28b-His Sumo vector via the restriction sites BamH1 and Xho1. The ligated plasmid was then transformed into *E*. *coli* BL21(DE3) cells. The resulting strain was grown to mid-log phase and then induced with 0.1 mM IPTG at 16°C for 16 h. Cells were collected by centrifugation and the pellet was resuspended in lysis buffer (50 mM Tris-HCl, pH8.0, 400 mM NaCl, 10% glycerol, 2 mM 2-mercaptoethanol and protease inhibitor). Resuspended cells were lysed by sonication and cleared by high speed centrifugation at 40,000 *g* for 1 h. The protein was purified using Ni-NTA agarose beads (GE), followed by enzyme digestion with Ulp1 to remove the His-Sumo tag. The digested product was further purified by gel-filtration chromatography (Hiload Superdex 75) and passed through Ni-NTA beads again to remove residual His-Sumo contamination. The buffer for gel-filtration chromatography buffer contains 25 mM Tris-HCl, pH8.0, 150 mM NaCl. The purified protein was concentrated to 22 mg/ml and store at -80°C.

### Crystallization and structural determination

Crystal screenings were performed with Hampton screening kits by sitting-drop-vapor-diffusion at 20°C. Trxlp was crystallized in precipitant/well solutions: 30% PEG-8000, 100 mM sodium acetate, pH 6.5, 200 mM lithium sulfate. All crystals were gradually transferred into harvesting solutions (precipitant solution and 25% glycerol) before being flash-frozen in liquid nitrogen. Datasets were collected under cryogenic conditions (100 K) at the Shanghai Synchrotron Radiation Facility (SSRF) beamlines BL19U1, and were processed by HKL3000_ENREF_22 [45]. The crystal belongs to space group *P6*122 with cell dimension a = b = 34.263 Å, c = 264.649 Å. There is one molecule in an asymmetrical unit. The structure was solved by MR_Rosetta in Phenix package using *Thermus thermophilus* thioredoxin (PDB:2YZU) as the initial model. Iterative cycles of refinement and modeling were carried out using Phenix [45] and Coot [46]. All the crystal structural figures were generated using PyMOL [47]. The coordinates have been deposited in the RCSB PDB under the code PDB: 5ZF2.

### Multiple sequence alignment

Acquiring sequences from NCBI databanks and building alignment by manual adjustments based on structural alignments generated by the DNAman server.

### Generation of ASK1-KO HeLa cells using CRISPR/Cas9

A CRISPR/Cas9 gRNA expression vector (pSpCas9(BB)-2A-Puro, #48138) was obtained from Addgene. The ASK1-KO target sequences were 5′-CACCGGCCGGG CAGCTTCTGGAACG-3′, and 5′-AAACCGTTCCAGAAGCTGCCCGGCC-3′. To generate ASK1-KO HeLa cell lines, DNA-In CRISPR Transfection Reagent (MTI-GlobalStem) was used to transfect the plasmids. Two to three days later, anti-puromycin cells were screened and cultured in DMEM complete medium containing 2 μg/ml puromycin. The puromycin resistant cells were series diluted and seeded into the 96-well plate. The ASK1-KO single clones were identified by immunoblotting with anti-ASK1 antibody.

### Preparation of macrophage-released *E*. *piscicida*

J774A.1 cell was infected as described previously with slight adjustments [24]. Briefly, *E*. *piscicida* EIB202 was grown overnight in tryptic soy broth (TSB) at 30°C with shaking and then diluted into fresh TSB with shaking at 30°C until the optical density at 600 nm reached 0.8. Harvested bacteria in phosphate-buffered saline (PBS) suspensions were added to macrophage cells at a multiplicity of infection (MOI) of 10:1 in DMEM containing 10% (vol/vol) FBS (growth medium, GM). Plates were then centrifuged at 600 *g* for 10 min, and gentamicin (100 μg/ml) was added 2 h after infection for 30 min to kill extracellular bacteria, followed by GM containing 10 μg/ml gentamicin for the remainder of the experiment at 30°C incubator. At indicated time points, the supernatant containing released bacteria was harvested. The supernatant was centrifuged at 600 *g* for 5 min to discard the cellular debris, and the harvested supernatant was further centrifuged at 13,000 *g* for 10 min to collect the macrophage-released bacteria. DMEM-cultured *E*. *piscicida* EIB202 was prepared as negative control.

### RNA extraction and quantitative real-time PCR

The macrophage-released and DMEM-cultured bacteria were prepared as described above [21]. RNA of both samples was extracted by using an RNA isolation kit (Tiangen, Beijing, China). One microgram of each RNA sample was used for cDNA synthesis with the FastKing One Step RT-PCR Kit (Tiangen) and quantitative real-time PCR (RT-qPCR) was performed on an FTC-200 detector (Funglyn Biotech, Shanghai, China) by using the SuperReal PreMix Plus (SYBR Green) (Tiangen). The gene expression of bacterial Trxlp was performed for three biological replicates, and the data for each sample were expressed relative to the expression level of the 16S gene by using the 2^-ΔΔCT^ method. The gene expression of TNF-α, IL-10, IL-6, IL-8 and IFN-γ genes was performed for three biological replicates, and the data for each sample were expressed relative to the expression level of the β-actin gene by using the 2^-ΔΔCT^ method.

### Secretion assay of Trx family proteins

For Trx family protein secretion analysis, wild type and Trx-HA family protein-expressing *E*. *piscicida* EIB202 strains were grown overnight in TSB medium and subcultured 1:100 in fresh DMEM and grow for an additional 15 h. L-Arabinose was added to induce the expression of Trx-1, Trx-2 and Trxlp-HA when OD_600_ was 0.6. To ensure that protein from equal numbers of cells was analyzed, protein samples were adjusted to a volume in which 1 ml of culture corresponds to OD_600_ = 1. Bacteria were collected in 50 ml tubes, and centrifuged at 5,000 *g* for 10 min at 4°C. Extracellular proteins were obtained by ultrafiltration from supernatants, which were filtered through a 0.22 μm filter membrane unit (Millipore, Darmstadt, Germany) with a 10 kDa molecular weight cut-off Amicon Ultra-15 centrifugal filter device (Millipore). 150 micrograms of protein were boiled for 10 min in SDS sample buffer before each protein mixture was subjected to SDS-PAGE or stored at -20°C before Western blot analysis. To explore the secretory manner of protein, wild-type, ΔT3SS and ΔT6SS *E*. *piscicida* overexpressing with Trxlp-HA plasmid were cultured as indicated above. The protein samples were prepared and determined in the same method described above.

### Isolation and analysis of *E*. *piscicida* OMVs

OMVs were fractionated by density gradient ultracentrifugation with OptiPrep (Sigma) as described [26]. Briefly, EIB202 containing Trxlp-HA plasmid were grown overnight in TSB medium and then subcultured 1:100 in fresh DMEM and grown for an additional 12 h. Bacteria were removed by centrifugation (5,000 *g*, 10 min, 4°C) and the DMEM supernatants were filtered through a 0.45 μm filter membrane unit (Millipore) with a 10 kDa molecular weight cut-off Amicon Ultra-15 centrifugal filter device. OMVs were collected from the filtered supernatants by ultracentrifugation (284,000 *g*, 1.5 h, 4°C) in a CP-RX80 (Hitachi, Japan) and the OMV pellets were resuspended in 100 μl PBS, OMV-free supernatants were obtained from OMV supernatants. The protein samples were prepared and determined in the same method described above. The amounts of OMV were identified by the anti-OmpA antibody, and anti-RNAP antibody (RNA polymerase) was used to detect the bacteria.

### Fractionation assay of *E*. *piscicida* infected HeLa cells

Infected HeLa cells were fractionated as reported previously with minor modifications [24]. Briefly, HeLa cells were seeded on 6-well/24-well culture dish at (35–40)×10^4^/(7.5–10)×10^4^ per well before infection. Overnight cultured *E*. *piscicida* strains were diluted 1:100 into fresh DMEM with Amp and Col antibiotics, grown for 9–12 h, and then added into HeLa cells at a MOI of 100 in DMEM with arabinose, and incubated for 3 h. The culture dishes were then washed three times with pre-warmed PBS, and the medium was replaced with pre-warmed DMEM with 5% FBS (with arabinose) and 10 μg/ml gentamicin for another 5 h. The supernatant and cytosolic fraction from infected HeLa cells was harvest and analyze as described [24].

### Immunofluorescence microscopy analysis of Trxlp translocation

Wild-type *E*. *piscicida* were electroporated with pUTt0456-Trxs-HA plasmids to express Trxs-HA protein constitutively. HeLa cells were seeded onto 24-well plates containing sterile coverslips and cultured overnight. Following infection with above strains and incubation with gentamicin, the cells were washed with PBS and then fixed in 4% (wt/vol) paraformaldehyde for 10 min at room temperature. Fixed cells were washed in PBS and permeabilized with 0.1% Triton X-100 for 5 min at room temperature. After being washed with PBS, blocking of non-specific binding was achieved by placing the coverslips in the fetal bovine serum (FBS) for 20 min at room temperature. After blocking, a dilute solution of anti-HA antibody (Molecular Probes) was incubated with the coverslips under 4°C overnight. After rinsing the membrane to remove unbounded primary anti-HA antibody, the coverslips were exposed to secondary antibody for one hour and the nuclei were stained with 4,6-diamidino-2-phenylindole (DAPI; Sigma) for 10 min at room temperature. Fixed samples were viewed on a Nikon A1R confocal microscope, and the images were analyzed using ImageJ (NIH).

### TEM1 protein translocation assay

The translocation of translational protein fusions between TEM1 and Trxlp were evaluated by the detection of β-lactamase activity in infected HeLa cells as previously described [22]. Briefly, TEM1 fusions (pCX340-*trx1*, -*trx2*, or -*trxlp*) were introduced into wild-type, ΔT3SS or ΔT6SS *E*. *piscicida* by electroporation. Bacteria were grown in TSB overnight at 30°C, then diluted into DMEM and grown standing at 30°C until OD_600_ reached 0.8. HeLa cells were then infected with strains harbouring the TEM1 fusions at a MOI of 100. Infected cells were centrifuged at 400 *g* for 10 min to initiate bacterial-cell contact followed by incubation at 35°C for 3 h after which the cells were washed 3 times and incubated with fresh DMEM without serum for another 4 h. At this time point, cells were washed three times with DMEM and loaded with the fluorescent substrate CCF2/AM (LiveBLAzer-FRET B/G loading kit; Invitrogen) in the β-lactamase loading solution supplemented with 15 mM Probenecid (Invitrogen). Cells were incubated in dark for 120 min at room temperature and then observed under a Nikon A1R confocal microscope.

### Co-immunoprecipitation of ASK1-TBD and Trxlp

ASK1-TBD (sequence 88–302) fragment was amplified from mammalian cells and identified by sequencing. HA-tagged ASK1-TBD expression plasmids in pCDH vector were constructed by introducing an HA epitope sequence at the C-terminal of ASK1-TBD by homologous recombination PCR (Vazyme, Product code C112). A GFP tag was inserted at the C-terminus of Trxlp in pCDH by PCR. HA-tagged ASK1-TBD (4 μg) and GFP-Trxlp (6 μg) expressing vectors were cotransfected into HEK293T cells in 10 cm dish with 10 ml medium by calcium phosphate transfection standard procedure. For immunoprecipitation, cells were lysed on ice using cell lysis buffer containing 20 mM Tris, 100 mM KCl, 0.1% NP-40, 1 mM EDTA, 10% glycerol, 10 mM tetrasodium pyrophosphate, fresh cocktail. The lysates were divided and incubated with HA beads, and 6 h later, HA beads were washed with cell lysis and wash buffer (20 mM Tris, 150 mM KCl, 0.5% NP-40, 1 mM EDTA, 1 ml EDTA, 10% glycerol, 10 mM tetrasodium pyrophosphate and fresh cocktail) each for three times. Then, the beads were immunoblotted with either anti-GFP or anti-HA antibody. The proteins were detected with the ECL system.

ASK1-N terminal (sequence 1–400) fragment was amplified from mammalian cells and identified by sequencing. HA- and Flag-tagged ASK1-N terminal expression plasmids in pCDH vector were constructed by introducing the tag sequence at the C-terminal of ASK1-N terminal by homologous recombination PCR (Vazyme, Product code C112). The ASK1-N-HA, ASK1-N-Flag and Trxlp-HA were transfected into HEK293T cells as above. Thirty-six hours post transfection, the homogenate was prepared for the immunoprecipitation assay. The beads were immunoblotted with either anti-Flag or anti-HA antibody. The proteins were detected with the ECL system.

### Expression and purification of Trxlp and ASK1-TBD

DNA encoding N-terminal fragments of human ASK1 (residues 88–302) were ligated into pET28a using the NcoI and XhoI sites, and 6 Histidine sequence was added by design of primers for proteins purification. Trxlp cloned from *E*. *piscicida* DNA was expressed with MBP tag (in pET28a) in *E*. *coli*. The recombinant tagged-fusion protein expression was induced by IPTG at 16°C for 16 h and purified from *E*. *coli* BL21 (DE3) cells. rASK1-TBD-MBP protein was washed with MBP binding buffer (50 mM Tris-HCl (pH 8.0), 400 mM NaCl, 5 mM DTT, 10% (w/v) glycerol), eluted against MBP binding buffer containing 20 mM maltose and purified using Amicon Ultra centrifugal filters (UFC501008, Amicon Ultra-0.5 Centrifugal Filter Unit with Ultracel-10 membrane). Trxlp-His protein was gradiently eluted with His binding buffer (Na_2_HPO_4_ (1.4 g/L), NaH_2_PO_4_ (1.216 g/L), NaCl (29.2 g/L), 5 mM DTT, imidazole), and Trxlp-his protein in 200 mM imidazole were concentrated using Amicon Ultra centrifugal filters (UFC501008, Amicon Ultra-0.5 Centrifugal Filter Unit with Ultracel-10 membrane) by centrifugation (≤ 5,000 *g*, 4°C). All ASK1-TBD mutants and Trxlp mutants were generated by PCR and mutations were confirmed by sequencing. The indicated mutant proteins were expressed and purified as described above.

### Enzymatic assay of thioredoxin proteins

The free thiol groups of purified Trx1, Trx2 and Trxlp of *E*. *piscicida* were analyzed by Ellman’s detection with DTNB according to previous study [28]. The indicated thioredoxin’s catalytic reduction experiment was performed according to previous studies with slightly changes [29–30]. Briefly, the increase in turbidity at 650 nm is plotted against the reaction time. The assay mixtures contained 170 uM insulin, 2 mM DTT in 50 mM Tris-HCl (pH 7.4), 1 mM EDTA (pH 7.0), and the same concentrations of Trx protein, or ddH_2_O as negative control.

### *In vitro* pulldown assay

Purified ASK1-His and Trxlp-MBP proteins were determined with Bradford standard method. Purified MBP fusion proteins immobilized on the MBP beads were incubated on ice for 30 min, and washed with MBP binding buffer for three times. Then ASK1-TBD-His protein was added into prepared Trxlp-MBP beads, incubated for another 4 h at 4°C, washed with MBP binding buffer to get rid of protein impurity, and finally eluted with MBP binding buffer containing 20 mM maltose. The eluent was detected with SDS-PAGE. Similarly, purified Trxlp His fusion proteins immobilized on His beads were incubated on ice for 30 min, washed with His binding buffer for three times. then Trxlp-MBP protein was added into prepared rASK1-TBD-His beads, incubated for another 4 h at 4°C, washed with His binding buffer containing 100 mM imidazole to remove the unbinding proteins, and finally eluted with His binding buffer containing 200 mM imidazole. The eluent was detected with SDS-PAGE. The binding complexes were determined by SDS-PAGE, and the lower part of the SDS-PAGE was cut out and probed with anti-His antibody or anti-MBP antibody. The protein was detected with the ECL system.

### MAPK pathway activation analysis

HeLa cells were seeded at a density of 5 × 10^6^ cells per well in 12-well plates and cultured overnight. Before infection, the culture medium was changed to serum-free DMEM for 12–16 h at 30°C. ZF4 cells were seeded at a density of 5×10^6^ cells per well in 12-well plates and cultured overnight. Before infection, the culture medium was changed to serum-free DF12 for 12–16 h at 28°C. Wild-type *E*. *piscicida* and indicated mutant strains were cultured as described above, and infected at an indicated MOI. At indicated time, the infected cells were detected by immunoblotting or RT-PCR.

HEK293T cells were transfected using standard calcium phosphate method [48], and the medium were changed to serum-free DMEM for 12–16 h before TNF-α stimulation.

### Western blot and antibodies

Cells were lysed in 50 mM Tris, 150 mM NaCl, 1% Triton X-100, and 1 mM EDTA with pH 7.4. Lysates were mixed with protein loading buffer, boiled, and centrifuged. 10 μl of the cell lysate was separated by SDS-PAGE on a 12% gel and transferred to PVDF membrane (Millipore), then probed with specific antibodies, and antibody binding detected by chemiluminescence. Antibodies for HA (0906–1), Flag (M1403-2) and β-actin (M1210-2) were purchased from HUABIO at 1:5000. Antibody for RNAP (sc-101597) was purchased from Santa Cruz at 1:1000. Tubulin (AF1216) and calnexin (AC018) antibodies were obtained from Beyotime Biotechnology at 1:1000. Anti-His antibody (AB102‐02; TianGen Biotech) or anti-MBP antibody (A00190‐100; Genscript Technology) was used as primary antibodies for immunoblotting. Antibodies for IκBα (4814), Erk1/2 (9107), phospho-Erk1/2 (9101s), p38α (9218), phospho-p38α (9215s), JNK (9252), phospho-JNK (9251S), phospho-MEK1/2 (9121), phospho-MKK3/6 (9231S), phospho-MKK4 (9156S), phospho-MKK7 (4171S) and phospho-ASK1 (3765) were purchased from Cell Signaling Technology at 1:1000. ASK1 antibody (ET1608-54) was from HUABIO at 1:1000. HRP-conjugated goat anti-mouse (A0216) and anti-rabbit IgG (A0208) antibodies were purchased from Beyotime Biotechnology at 1:5000.

### MO design and analysis

An MO (Gene Tools) was designed to target a site in *ask1* to block its translation (5’- TGAAGCGACACTCAGCAGTAGCTG-3’), and a corresponding 5-base mismatch oligonucleotide was used as a specificity control (5’-TcAAcCGACAgTCAGCAcTAGgTG-3’). Embryos were injected as described above. MOs were used at 0.25, 0.5, and 1 ng per embryo, and the results of experiments using three batches of embryos were analyzed independently. Knockdown of *ask1* was verified by immunoblotting with anti-ASK1 antibody.

### Zebrafish larvae microinjection infection model

Zebrafish embryos were collected from a laboratory-breeding colony kept at 28°C on a 12:12 h light/dark rhythm as previously described [37]. Embryos were raised in Petri dishes with E3 medium (5 mM NaCl, 0.17 mM KCl, 0.33 mM CaCl_2_, 0.33 mM MgSO_4_) containing 0.3 μg/ml methylene blue at 28°C. Zebrafish larvae were maintained until 3-day post fertilization (dpf), at which time all were euthanized by 4 g/L buffered tricaine (MS-222, ethyl 3-aminobenzoate methanesulfonate, Sigma-Aldrich) in accordance with ethical procedures. For microinjection experiments, the bacteria were prepared as described in culture conditions. Injections were done using pulled borosilicate glass microcapillary injection needles (Sutter) and a Milli-Pulse Pressure Injector (ASI). Prior to injections, embryos of 3 dpf were manually dechorionated and anesthetized with 200 mg/L buffered tricaine (MS-222). Afterwards embryos were aligned on an agar plate and injected with 1 nl of the indicated *E*. *piscicida* suspension into the yolk sac. Prior determination of the injected volume was performed by injection of a droplet into mineral oil and measurement of its approx. diameter over a scale bar. After injections, infected larvae were allowed to recover in a Petri dish with fresh E3 medium for 15 min. Subsequently, larvae were transferred in 10 cm dish in groups of about 50 larvae in 15 ml E3 medium per dish, incubated at 28°C. The mortality rate and bacterial colonization at different time points were observed. The RNA of infected zebrafish larvae was extracted, and the expression of TNF-α, IL-10, IL-6, IL-8 and IFN-γ were detected by RT-PCR as described above.

### Statistical analysis

Statistical analysis was performed using GraphPad Prism program (GraphPad Software). All data were representative of at least three independent experiments and were presented as mean ± SD (standard deviation). Western bolts were analyzed by Quantity One Software, and Gauss model trace of each bands was calculated based on the equation Band-Gauss Model Bands. Differences between two groups were evaluated using Student’s *t* test. One-way ANOVA test was used to analyze differences among multiple groups. Differences in larvae survival were assessed using the log-rank (Mantel-Cox) test. Statistical significance was defined as * *p*<0.05, ** *p*<0.01, *** *p*<0.001.

## Supporting information

S1 Fig The diagram of the antioxidant system in E. piscicida. Thioredoxin (Trx), GSH and catalase are present in EIB202. The Trx antioxidant system in the bacterium contains one TrxR, three Trxs (Trx1, Trx2, and Trxlp), and three major thiol peroxidases (Bcp, Tpx, and Msr). Transcripts of Trx family genes were detected in macrophage-released and DMEM-cultured EIB202 by real-time PCR and the fold changes after infection are indicated. Transcript expression of 16S RNA was used as an internal control. Data are representative of at least 3 experiments.
(TIF)

S2 Fig Assay of intracellular translocation of Trxlp by immunofluorescence. HeLa cells were infected with Trxs-HA fusion-expressing EIB202 at a MOI of 100. The translocation of Trxlp-HA was examined by immunofluorescence using anti-HA antibody. Green indicates positive HeLa cells. Percentages of cells positive for intracellular Trxlp are listed below (approximately 200 cells were counted in each sample. Means ± SD of triplicate samples).
(TIF)

S3 Fig Superimposition of thioredoxin from different species. Trxlp (PDB:5ZF2), green; *Homo sapiens* thioredoxin 1 (PDB: 1ERT), yellow; *Homo sapiens* thioredoxin 2 (PDB:1UVZ), cyan; *Thermus thermophilus* thioredoxin 1 (PDB: 2YZU), red; *E*. *coli* thioredoxin 1 (PDB:2TRX), orange.
(TIF)

S4 Fig Enzymatic activities of Trxlp and related classical Trx proteins from E. piscicida. (A) Schematic representation of Trx1, Trx2, and Trxlp. The red circles represent the conserved CXXC-motif domain, and the mitochondrial targeting sequence is marked by a blue line. (B) Ellman’s detection of the free thiol groups of thioredoxin with DTNB. DTNB is reduced by SH groups to form 1 mole of 2-nitro-5-mercaptobenzoic acid per mole of SH. (C) Thioredoxin-catalyzed reduction of insulin by DTT. The increase in turbidity at 650 nm is plotted against the reaction time. (B-C) Data are representative of at least 3 experiments.
(TIF)

S5 Fig The effects of Trxlp in regulating MAP2K and MAPK signaling. (A) Wild-type HeLa cells were infected with EIB202, *Δtrx1*, *Δtrx2*, or *Δtrxlp E*. *piscicida* at a MOI of 100 for indicated time points, and the cell lysates were probed for anti-phospho-Erk1/2, anti-Erk1/2, anti-phospho-p38, anti-p38, anti-phospho-JNK, anti-JNK and anti-IκBα antibodies. (B) HEK293T cells transfected with Trxlp-HA or human TRX1-HA expression vectors were pretreated with TNF-α. Cell lysates were probed with anti-phospho-Erk1/2, anti-Erk1/2, anti-phospho-p38, anti-p38 and anti-HA antibodies. (C) HEK293T cells transfected with wild-type Trxlp (WCXXC)-HA or mutant Trxlp (FSXXS)-HA expression vectors were pretreated with TNF-α. Cell lysates were probed with anti-phospho-Erk1/2, anti-Erk1/2, anti-phospho-p38, anti-p38 and anti-HA antibodies. (D) Wild-type HeLa cells and ASK1-KO HeLa cells were infected with wild-type (EIB202), *Δtrxlp*, or *trxlp*-complemented *E*. *piscicida* at a MOI of 100 for 2 hours. The cell lysates were probed for anti-phospho-JNK and anti-JNK, anti-phospho-MKK7 and anti-MKK7 antibodies. (A-D) β-Actin is shown as a loading control. The signal intensities were quantitatively analyzed using Quantity one software. Data are representative of at least 3 experiments.
(TIF)

S6 Fig The effects of Trxlp in regulating MAP2K signaling and inflammatory cytokine expression in ZF4 cells during E. piscicida infection. (A) ZF4 cells were infected with EIB202, *Δtrxlp*, or *trxlp*-complemented *E*. *piscicida* at a MOI of 10 for 2 H, as shown in Fig 4D. Cell lysates were probed with anti-phospho-MEK1/2 and anti-MEK1/2, anti-phospho-MKK3/6 and anti-MKK3/6, anti-phospho-MKK4 and anti-MKK4, anti-phospho-MKK7 and anti-MKK7, and anti-β-actin antibodies. Data are representative of at least 3 experiments. (B-F) mRNA levels of TNF-α, IL-10, IL-6, cxcl8 and IFN-γ in ZF4 cells infected with EIB202, *Δtrxlp*, or *trxlp*-complemented *E*. *piscicida* at indicated time points were measured by qRT-PCR. Data (mean ± SD) shown are from three representative experiments. * *p* < 0.05. (G) The protein expression level of TNF-α in ZF4 cells infected with EIB202, *Δtrxlp*, or *trxlp*-complemented *E*. *piscicida* at the indicated time points were measured by ELISA assays. Data (mean ± SD) shown are from three representative experiments. * *p* < 0.05.
(TIF)

S7 Fig ASK1 knockdown in zebrafish larvae and inflammatory cytokine expression during E. piscicida infection in vivo. (A) Immunoblotting assay of ASK1 expression from 3 and 7 day-post fertilization (dpf) *ask1*-morphants and control-larvae. *n* = 5 fish per sample at each time point. (B-D) mRNA levels of *IL-6*, *cxcl8* and *IFN-γ* in indicated zebrafish larvae infected with EIB202, *Δtrxlp*, or *trxlp*-complemented *E*. *piscicida* at indicated time points were measured by qRT-PCR as in Fig 5D. Data (mean ± SD) shown are from 3 representative experiments.
(TIF)

S1 Table Top 10 up-regulated genes annotated in macrophage-released E. piscicida. Functional annotation of the top 10 up-regulated genes in macrophage-released EIB202 compared with DMEM-cultured EIB202 based on RNA-seq data [24]. Read_C, the reads count in control group; Read_T, the reads count in EIB202 infection group; RPM, reads of exon model per million mapped reads.
(XLSX)

S2 Table X-ray diffraction data-collection and structure-refinement statistics. (XLSX)

S3 Table Statistical analysis of Fig 5C. Results are representative of three separate experiments. *p < 0.05, **p < 0.01, *** *p* < 0.001 by unpaired two-tailed Student’s *t* test; ns = not significant.
(XLSX)

S4 Table Strains and plasmids used in this study. (XLSX)

S5 Table Primers used in this study. (XLSX)
