# Supplementary figures and images for: The Edwardsiella piscicida thioredoxin-like protein inhibits ASK1-MAPKs signaling cascades to promote pathogenesis during infection
Source: PLoS Pathog. 2019 Jul 17;15(7):e1007917. doi: 10.1371/journal.ppat.1007917 (PMC6636751; doi:10.1371/journal.ppat.1007917)

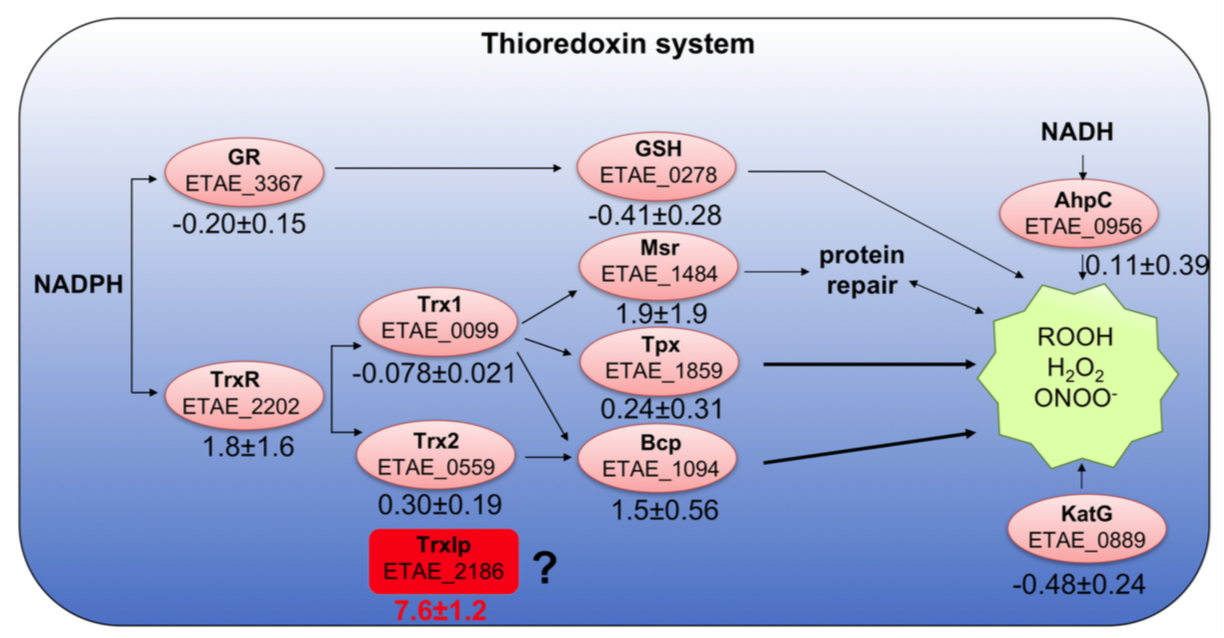

Supplement: S1 Fig — Thioredoxin (Trx), GSH and catalase are present in EIB202. The Trx antioxidant system in the bacterium contains one TrxR, three Trxs (Trx1, Trx2, and Trxlp), and three major thiol peroxidases (Bcp, Tpx, and Msr). Transcripts of Trx family genes were detected in macrophage-released and DMEM-cultured EIB202 by real-time PCR and the fold changes after infection are indicated. Transcript expression of 16S RNA was used as an internal control. Data are representative of at least 3 experiments. (TIF) [file ppat.1007917.s001.tif]

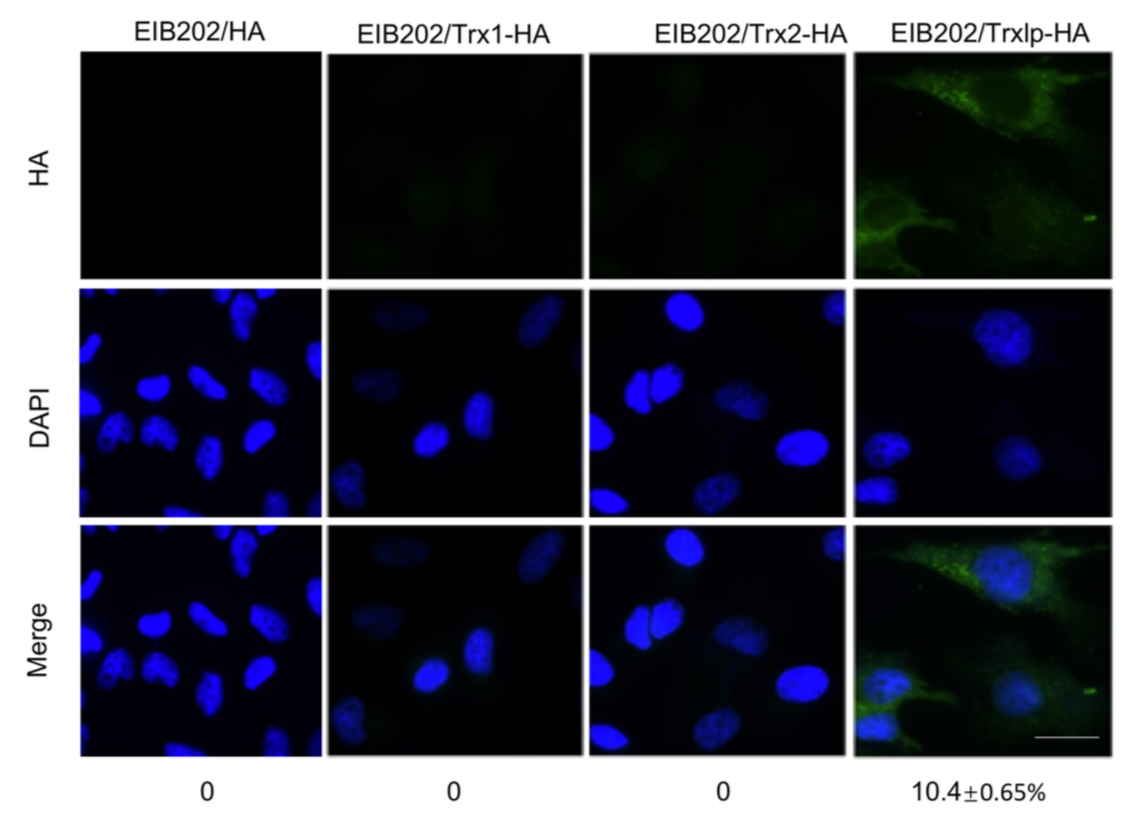

Supplement: S2 Fig — HeLa cells were infected with Trxs-HA fusion-expressing EIB202 at a MOI of 100. The translocation of Trxlp-HA was examined by immunofluorescence using anti-HA antibody. Green indicates positive HeLa cells. Percentages of cells positive for intracellular Trxlp are listed below (approximately 200 cells were counted in each sample. Means ± SD of triplicate samples). (TIF) [file ppat.1007917.s002.tif]

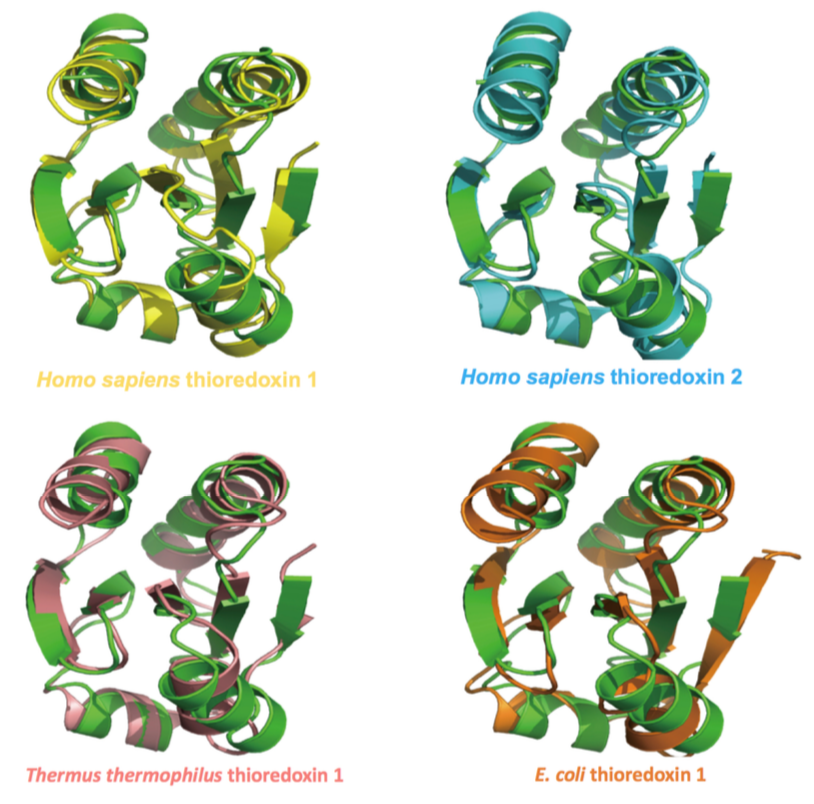

Supplement: S3 Fig — Trxlp (PDB:5ZF2), green; Homo sapiens thioredoxin 1 (PDB: 1ERT), yellow; Homo sapiens thioredoxin 2 (PDB:1UVZ), cyan; Thermus thermophilus thioredoxin 1 (PDB: 2YZU), red; E. coli thioredoxin 1 (PDB:2TRX), orange. (TIF) [file ppat.1007917.s003.tif]

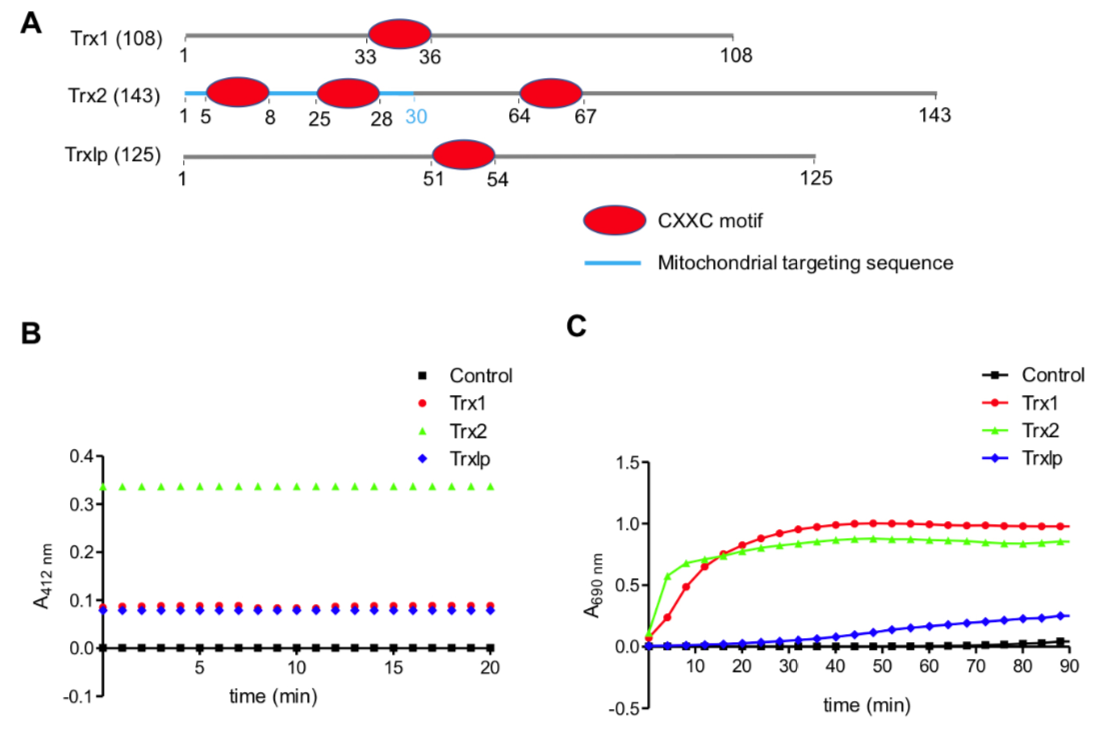

Supplement: S4 Fig — (A) Schematic representation of Trx1, Trx2, and Trxlp. The red circles represent the conserved CXXC-motif domain, and the mitochondrial targeting sequence is marked by a blue line. (B) Ellman’s detection of the free thiol groups of thioredoxin with DTNB. DTNB is reduced by SH groups to form 1 mole of 2-nitro-5-mercaptobenzoic acid per mole of SH. (C) Thioredoxin-catalyzed reduction of insulin by DTT. The increase in turbidity at 650 nm is plotted against the reaction time. (B-C) Data are representative of at least 3 experiments. (TIF) [file ppat.1007917.s004.tif]

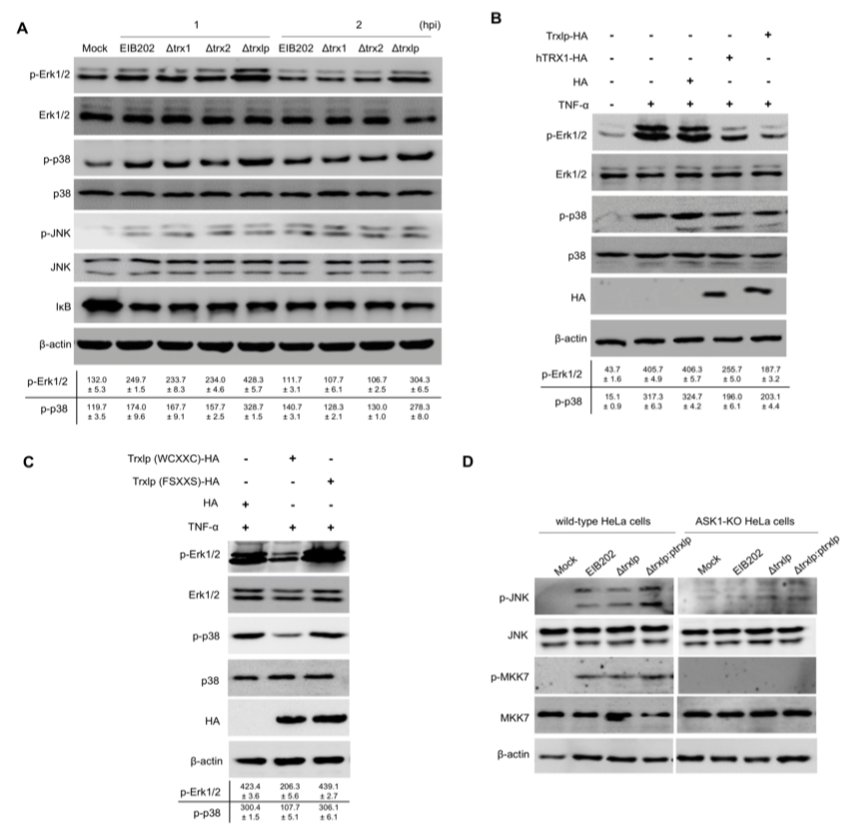

Supplement: S5 Fig — (A) Wild-type HeLa cells were infected with EIB202, Δtrx1, Δtrx2, or Δtrxlp E. piscicida at a MOI of 100 for indicated time points, and the cell lysates were probed for anti-phospho-Erk1/2, anti-Erk1/2, anti-phospho-p38, anti-p38, anti-phospho-JNK, anti-JNK and anti-IκBα antibodies. (B) HEK293T cells transfected with Trxlp-HA or human TRX1-HA expression vectors were pretreated with TNF-α. Cell lysates were probed with anti-phospho-Erk1/2, anti-Erk1/2, anti-phospho-p38, anti-p38 and anti-HA antibodies. (C) HEK293T cells transfected with wild-type Trxlp (WCXXC)-HA or mutant Trxlp (FSXXS)-HA expression vectors were pretreated with TNF-α. Cell lysates were probed with anti-phospho-Erk1/2, anti-Erk1/2, anti-phospho-p38, anti-p38 and anti-HA antibodies. (D) Wild-type HeLa cells and ASK1-KO HeLa cells were infected with wild-type (EIB202), Δtrxlp, or trxlp-complemented E. piscicida at a MOI of 100 for 2 hours. The cell lysates were probed for anti-phospho-JNK and anti-JNK, anti-phospho-MKK7 and anti-MKK7 antibodies. (A-D) β-Actin is shown as a loading control. The signal intensities were quantitatively analyzed using Quantity one software. Data are representative of at least 3 experiments. (TIF) [file ppat.1007917.s005.tif]

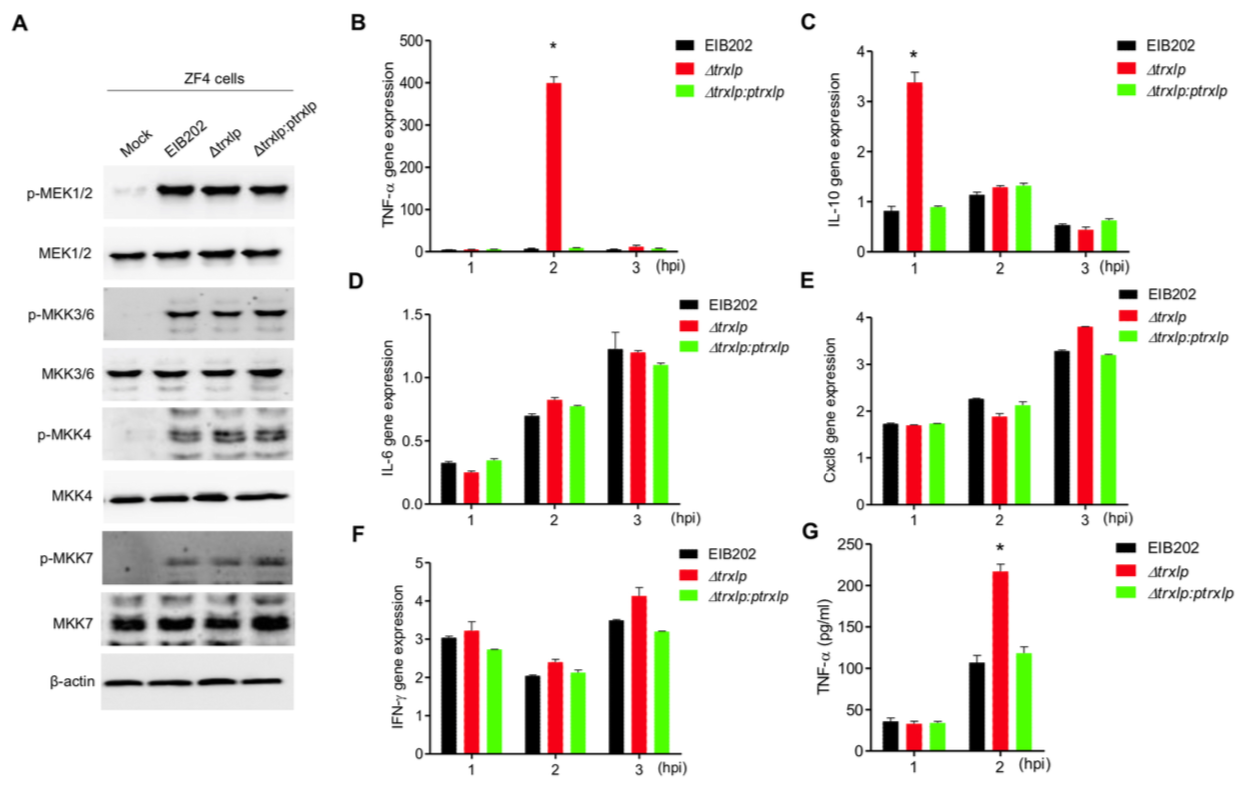

Supplement: S6 Fig — (A) ZF4 cells were infected with EIB202, Δtrxlp, or trxlp-complemented E. piscicida at a MOI of 10 for 2 H, as shown in Fig 4D. Cell lysates were probed with anti-phospho-MEK1/2 and anti-MEK1/2, anti-phospho-MKK3/6 and anti-MKK3/6, anti-phospho-MKK4 and anti-MKK4, anti-phospho-MKK7 and anti-MKK7, and anti-β-actin antibodies. Data are representative of at least 3 experiments. (B-F) mRNA levels of TNF-α, IL-10, IL-6, cxcl8 and IFN-γ in ZF4 cells infected with EIB202, Δtrxlp, or trxlp-complemented E. piscicida at indicated time points were measured by qRT-PCR. Data (mean ± SD) shown are from three representative experiments. * p < 0.05. (G) The protein expression level of TNF-α in ZF4 cells infected with EIB202, Δtrxlp, or trxlp-complemented E. piscicida at the indicated time points were measured by ELISA assays. Data (mean ± SD) shown are from three representative experiments. * p < 0.05. (TIF) [file ppat.1007917.s006.tif]

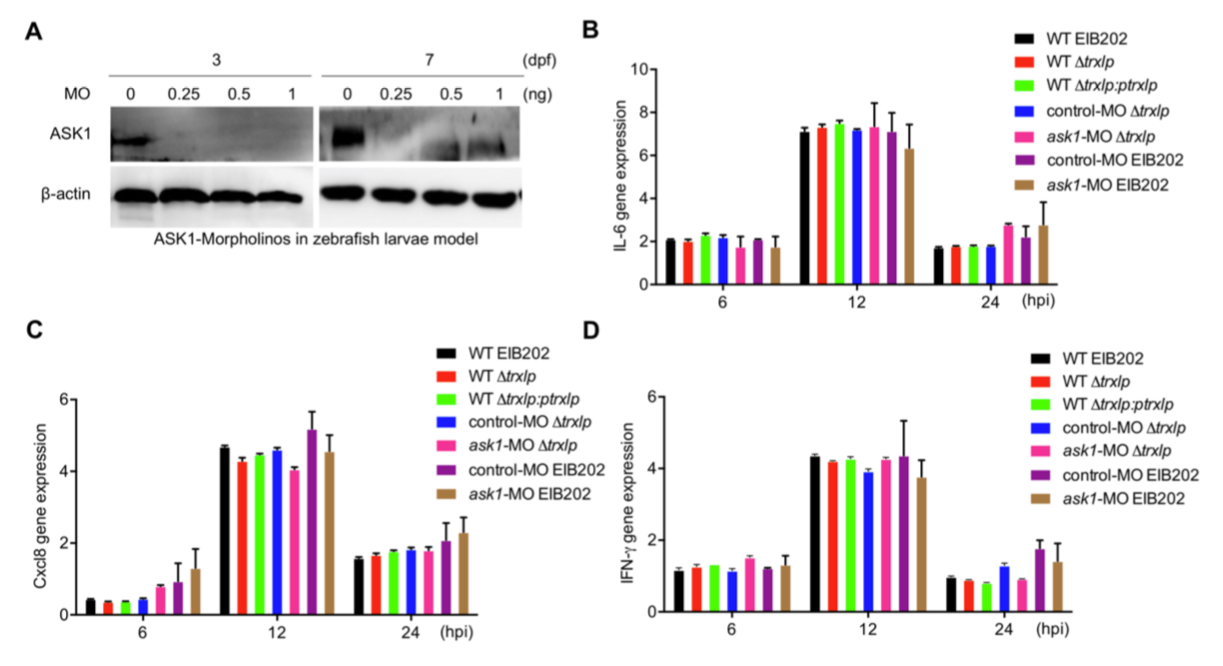

Supplement: S7 Fig — (A) Immunoblotting assay of ASK1 expression from 3 and 7 day-post fertilization (dpf) ask1-morphants and control-larvae. n = 5 fish per sample at each time point. (B-D) mRNA levels of IL-6, cxcl8 and IFN-γ in indicated zebrafish larvae infected with EIB202, Δtrxlp, or trxlp-complemented E. piscicida at indicated time points were measured by qRT-PCR as in Fig 5D. Data (mean ± SD) shown are from 3 representative experiments. (TIF) [file ppat.1007917.s007.tif]
